# OralDisk: A Chair-Side Compatible Molecular Platform Using Whole Saliva for Monitoring Oral Health at the Dental Practice

**DOI:** 10.3390/bios11110423

**Published:** 2021-10-28

**Authors:** Desirée Baumgartner, Benita Johannsen, Mara Specht, Jan Lüddecke, Markus Rombach, Sebastian Hin, Nils Paust, Felix von Stetten, Roland Zengerle, Christopher Herz, Johannes R. Peham, Pune N. Paqué, Thomas Attin, Joël S. Jenzer, Philipp Körner, Patrick R. Schmidlin, Thomas Thurnheer, Florian J. Wegehaupt, Wendy E. Kaman, Andrew Stubbs, John P. Hays, Viorel Rusu, Alex Michie, Thomas Binsl, David Stejskal, Michal Karpíšek, Kai Bao, Nagihan Bostanci, Georgios N. Belibasakis, Konstantinos Mitsakakis

**Affiliations:** 1 Hahn-Schickard, Georges-Koehler-Allee 103, 79110 Freiburg, Germany; Benita.Johannsen@Hahn-Schickard.de (B.J.); Mara.Specht@Hahn-Schickard.de (M.S.); Jan.Lueddecke@Hahn-Schickard.de (J.L.); Markus.Rombach@Hahn-Schickard.de (M.R.); sebastian.hin@iuvas.de (S.H.); Nils.Paust@Hahn-Schickard.de (N.P.); Felix.von.Stetten@Hahn-Schickard.de (F.v.S.); Roland.Zengerle@Hahn-Schickard.de (R.Z.); 2 Laboratory for MEMS Applications, IMTEK–Department of Microsystems Engineering, University of Freiburg, Georges-Koehler-Allee 103, 79110 Freiburg, Germany; 3 AIT Austrian Institute of Technology, Molecular Diagnostics, Giefinggasse 4, 1210 Wien, Austria; christopher_herz@pall.com (C.H.); Johannes.Peham@ait.ac.at (J.R.P.); 4 Clinic of Conservative and Preventive Dentistry, Center of Dental Medicine, University of Zurich, Plattenstrasse 11, 8032 Zurich, Switzerland; punenina.paque@zzm.uzh.ch (P.N.P.); thomas.attin@zzm.uzh.ch (T.A.); Joel.Jenzer@icloud.com (J.S.J.); philipp.koerner@zzm.uzh.ch (P.K.); patrick.schmidlin@zzm.uzh.ch (P.R.S.); Thomas.Thurnheer@zzm.uzh.ch (T.T.); florian.wegehaupt@zzm.uzh.ch (F.J.W.); 5 Department of Medical Microbiology and Infectious Diseases, Erasmus University Medical Centre Rotterdam (Erasmus MC), 3015 CN Rotterdam, The Netherlands; w.e.kaman@acta.nl (W.E.K.); j.hays@erasmusmc.nl (J.P.H.); 6 Department of Oral Biochemistry, Academic Centre for Dentistry Amsterdam (ACTA), Free University of Amsterdam and University of Amsterdam, 1081 LA Amsterdam, The Netherlands; 7 Department of Pathology and Clinical Bioinformatics, Erasmus University Medical Centre Rotterdam (Erasmus MC), 3015 CN Rotterdam, The Netherlands; a.stubbs@erasmusmc.nl; 8 Magtivio B.V., Daelderweg 9, 6361 HK Nuth, The Netherlands; vru@magtivio.com; 9 ClinicaGeno Ltd., 11 Station Approach, Coulsdon CR5 2NR, UK; alex@clinicageno.com (A.M.); thomas@clinicageno.com (T.B.); 10 Department of Biomedical Sciences, Faculty of Medicine, University of Ostrava, Syllabova 19, 70300 Ostrava, Czech Republic; david.stejskal@fno.cz; 11 Institute of Laboratory Diagnostics, University Hospital Ostrava, 17. Listopadu 1790/5, 70800 Ostrava, Czech Republic; 12 BioVendor-Laboratorní Medicína a.s., Research & Diagnostic Products Division, Karasek 1767/1, Reckovice, 62100 Brno, Czech Republic; karpisek@biovendor.com; 13 Faculty of Pharmacy, Masaryk University, Palackeho trida 1946/1, 61242 Brno, Czech Republic; 14 Section of Oral Health and Periodontology, Division of Oral Diseases, Department of Dental Medicine, Karolinska Institutet, 14104 Huddinge, Sweden; kai.bao@ki.se (K.B.); nagihan.bostanci@ki.se (N.B.); george.belibasakis@ki.se (G.N.B.)

**Keywords:** dental practice, point-of-care diagnostics, treatment monitoring, oral health, periodontitis, caries, saliva diagnostics

## Abstract

Periodontitis and dental caries are two major bacterially induced, non-communicable diseases that cause the deterioration of oral health, with implications in patients’ general health. Early, precise diagnosis and personalized monitoring are essential for the efficient prevention and management of these diseases. Here, we present a disk-shaped microfluidic platform (OralDisk) compatible with chair-side use that enables analysis of non-invasively collected whole saliva samples and molecular-based detection of ten bacteria: seven periodontitis-associated (*Aggregatibacter actinomycetemcomitans*, *Campylobacter rectus*, *Fusobacterium nucleatum*, *Prevotella intermedia*, *Porphyromonas gingivalis*, *Tannerella forsythia*, *Treponema denticola*) and three caries-associated (oral *Lactobacilli*, *Streptococcus mutans*, *Streptococcus sobrinus*). Each OralDisk test required 400 µL of homogenized whole saliva. The automated workflow included bacterial DNA extraction, purification and hydrolysis probe real-time PCR detection of the target pathogens. All reagents were pre-stored within the disk and sample-to-answer processing took < 3 h using a compact, customized processing device. A technical feasibility study (25 OralDisks) was conducted using samples from healthy, periodontitis and caries patients. The comparison of the OralDisk with a lab-based reference method revealed a ~90% agreement amongst targets detected as positive and negative. This shows the OralDisk’s potential and suitability for inclusion in larger prospective implementation studies in dental care settings.

## 1. Introduction

Oral diseases are the most prevalent chronic diseases worldwide, accounting for almost 5 billion cases globally [1]. They are the third most expensive group of diseases to treat in the EU, following diabetes and cardiovascular diseases [2]. In addition, the overprescription of antibiotics in dentistry is a challenge [3]. Indicatively, up to 80% of prophylactic antibiotic use in the US is considered unjustified [4].

The two most prevalent oral microbial diseases are caries and periodontitis. Dental caries affects the hard tissue of the teeth, causing tooth decay. Periodontitis affects the tissues that surround and support the teeth, leading to progressive loss of the bone and soft tissue attachment and eventually tooth loss. There are different clinical manifestations and degrees of severity of periodontal disease. According to the Global Burden of Disease (GBD) study, 796 million people around the globe had severe periodontitis in 2017, ranking it within the top 10 most prevalent conditions worldwide [5]. Across Europe, 5–20% of middle-aged people and up to 40% of elderly people are affected by it [6]. In the US, 80% of the population has some form of periodontal disease [7]. Periodontitis has also been related to systemic diseases such as type 2 diabetes mellitus (T2DM), cardiovascular diseases, Alzheimer’s disease and others [8,9,10]. Therefore, being able to detect periodontitis-causing bacteria has a much broader clinical significance than simply monitoring oral health, as it can potentially provide a signal for deteriorating systemic health [11].

Caries and periodontitis are both treatable, and the rationale around their treatment is the same, namely, the removal of the microbial biofilm which is the causative factor. The treatment of periodontal disease, upon the removal of the biofilm, requires good oral hygiene protocols and monitoring to ensure that the inflammation has subsided. For dental caries, upon removal of the infective caries tissue, the treatment mandates restoration of the lost hard tissue with appropriate restorative material (fillings). Although the treatment protocols for both diseases are well-defined, there exist urgent and unmet medical (dental) needs related to: (i) early diagnosis (even before symptoms emerge), which would assist in preventing the disease, benefiting the patients’ quality of life while also saving on the costs of treatment and (ii) accurate monitoring during and after treatment (i.e., during the maintenance phase of treatment) in cases where a patient presents with advanced or aggressive disease. Accurate monitoring would allow the dentist to make the correct assessment of when to start and finish treatment: not too early, running the risk of re-emergence, and not too late, thus spending unnecessary resources. 

The current state of the art for performing such diagnoses and monitoring is still largely dependent on clinical examination, patient history and radiographic imaging (X-rays). However, radiographs mainly observe damage that occurred in the past, while plain clinical examinations tend to miss the first signs of incipient dysbiosis and therefore do not contribute to early detection and prevention. Furthermore, cumulative X-ray radiation during periodontal treatment and frequent visits for follow-up monitoring poses health risks and special concerns in some patients (e.g., pregnant women). Periodontal probing is a frequently used methodology in which a probe is inserted into the gingival sulcus in order to measure pocket depths around a tooth and to assess the health status of the periodontium [12,13]. However, it is an invasive method in the gums, and as such, it should not be used in patients with T2DM as it may lead to bacteremia [14,15], thereby increasing systemic inflammation and the infection risk in already vulnerable T2DM patients. Additionally, general clinical examinations do not include probing of the whole gingival sulcus of the teeth. Other typical (simplified) measurements for basic periodontal screening include the community periodontal index (CPI) and the basic periodontal examination (BPE) [16], which are performed by looking for increased pocket depths and bleeding on probing. 

Unfortunately, these current ’gold-standard’ diagnostic approaches may risk missing the diagnosis of emerging periodontitis. Therefore, complementary diagnostic tools, which are largely less invasive and less tedious, could improve the diagnostic sensitivity, precision and accuracy, for example, by screening the microbial ecology of the oral cavity in order to help indicate whether a patient needs further diagnostic evaluation and treatment or to monitor patients’ post-treatment status. 

In this respect, the current publication proposes a rapid, molecular-based and non-invasive platform for detecting oral disease-causing bacteria, with a workflow that is compatible with point-of-care (POC) dental settings [17] and which can function as an auxiliary tool to the current gold standard diagnostic methods. The OralDisk microfluidic cartridge integrates all the biochemical reagents needed for fully automated analysis, namely: (i) customized buffers and microfluidic-optimized magnetic particles for the purification of bacterial DNA; (ii) amplification reagents in a lyophilized form; and (iii) POC-ready real-time qPCR TaqMan primers/probes for specific bacterial detection. The OralDisk is an application-specific version of the centrifugal microfluidic LabDisk platform, which has already demonstrated its utility in applications where a single infectious pathogen is to be detected [18,19,20,21]. This small-scale technical feasibility study demonstrates for the first time the platform’s implementation in the field of oral health, where multiple bacteria may be present simultaneously in the oral cavity and whole saliva is used as diagnostic specimen. It presents data on the detection of three caries-associated and seven periodontitis-associated bacterial species in complex saliva samples collected from individuals that were classified into three study groups (healthy, caries and periodontitis) following an assessment by dental specialists. The patients’ status assessment and the sample collection were performed in a previous clinical study [22]. The results from the OralDisk were compared with a lab-based extraction and qPCR reference method, as well as with the iai PadoTest (Institut für Angewandte Immunologie IAI AG, Zuchwil, Switzerland) commercial reference method. The OralDisk exhibited comparable (and in some cases superior) behavior while offering the additional benefit of automation.

## 2. Materials and Methods

### 2.1. Sample Collection and Ethics Permission

The samples used in this study were a sub-group (*n* = 24) of a large cohort (*n* = 214) of samples that had been collected at the Center for Dental Medicine, University of Zurich. These samples were intended for the microbial analysis of saliva with the aim of identifying oral infections in patients [22]. The 24 samples (seven healthy, nine caries and eight periodontitis samples) were selected in order to demonstrate the technical feasibility of the OralDisk, without the intention of generating clinical conclusions. The sample collection and the study protocol were approved by the local Swiss ethics committee (BASEC-no. 2016-00435) and all sample donors signed a written informed consent form prior to saliva collection. The collection contained unstimulated whole saliva that was aliquoted and stored at −80 °C until further analysis. Details on the inclusion/exclusion criteria and on the collection methodology are available in Paqué et al. [22].

### 2.2. Selected Bacterial Panel

Quantitative shifts in the levels of multiple, rather than single, bacterial species may more accurately reflect dysbiotic changes in oral microbial ecology that are commensurate with the initiation or progression of oral disease [23]. Measurement of the species dynamics of oral polymicrobial populations is the key element that allows potential monitoring for the prevention, early diagnosis and post-treatment follow-up of oral diseases. Therefore, ten bacteria were included in the OralDisk panel: seven Gram-negative bacteria related to periodontitis (*Aggregatibacter actinomycetemcomitans*, *Campylobacter rectus*, *Fusobacterium nucleatum*, *Prevotella intermedia*, *Porphyromonas gingivalis*, *Tannerella forsythia* and *Treponema denticola*) and three Gram-positive bacteria associated with caries (oral associated *Lactobacilli*, *Streptococcus mutans* and *Streptococcus sobrinus*). 

This panel was chosen based on: (i) current knowledge on the association of certain oral bacteria with caries and periodontitis [24]; (ii) feedback from experts in response to survey questionnaires; and (iii) the goal of including bacteria which were identified in subgingival [25] and supragingival [26] biofilm samples of subjects with and without periodontitis. Furthermore, the role of these bacteria as differentiators between healthy, periodontitis and caries groups was demonstrated in a preceding clinical study by Paqué et al. [22], which provided additional evidence for including these particular bacteria in the current technical feasibility study of the OralDisk. In the aforementioned clinical study, statistically significant polymicrobial differentiators were observed (i) between healthy and periodontitis groups (*C. rectus*, *T. forsythia*, *P. gingivalis*, *S. mutans*, *F. nucleatum*, *T. denticola*, *P. intermedia* and oral *Lactobacilli*); (ii) between healthy and caries groups (*S. mutans* and *T. denticola*); and (iii) between caries and periodontitis groups (*S. mutans*).

### 2.3. Reference Method #1: Lab-Based DNA Extraction and qPCR

**Figure 1:**
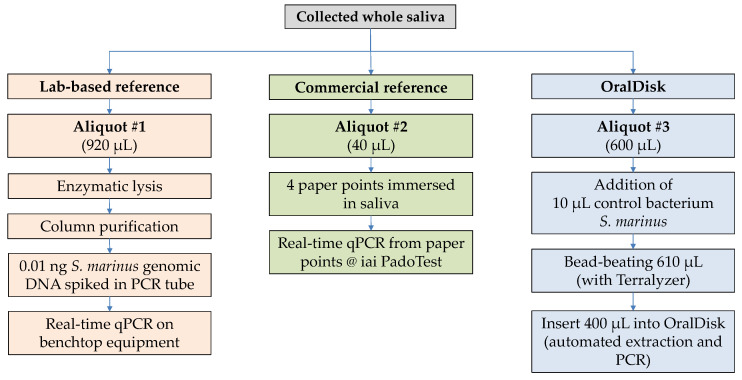
Experimental workflows from sample collection until analysis.

Enzymatic lysis was performed on 920 µL of whole saliva using the GenElute^TM^ Bacterial Genomic DNA Kit (Sigma-Aldrich, Saint Louis, MO, USA), followed by silica column-based extraction (Figure 1). An adjusted manufacturer’s protocol was used, as described in previous work [22]. An eluate volume of 135 µL was stored at −25 °C and later used to perform qPCR (Roche LightCycler). Details on the POC-compatible qPCR assay development, primer/probe design, assay validation (including qPCR assay sensitivity and limit of detection), utilized amplification conditions and data analysis, are available in Paqué et al. [22].

### 2.4. Reference Method #2: Commercial iai PadoTest

The iai PadoTest (Institut für Angewandte Immunologie IAI AG, Zuchwil, Switzerland) [27] is a commercially available test that was used as a second reference to the OralDisk (Figure 1). It performs a multiplex real-time qPCR assay that estimates bacterial cell counts based on 16S rRNA [28]. The manufacturer’s collection protocol stipulates that paper points are inserted into dental pockets to collect gingival crevicular fluid (GCF). The paper points are stored in vials (one or more paper points per vial are possible, in single- or pool-mode of analysis). To make the results comparable with our saliva-based detection, the sample collection protocol was slightly modified: four paper points (Roeko Iso 55, Coltène, Altstätten, Switzerland) were immersed in 40 µL of thawed whole saliva in a tube. The tubes with the paper points were sent to iai PadoTest AG for analysis.

### 2.5. Mechanical Lysis and Homogenization of Saliva Samples Prior to Insertion into the OralDisk

Lysis of bacteria, with simultaneous homogenization of the saliva, was performed prior to insertion into the OralDisk by means of a mechanical bead-beating process using a hand-held device (Terralyzer, Zymo Research, Irvine, CA, USA). Notably, this was the only manual step in the protocol (Figure 1). A volume of 600 µL of whole saliva and 10 µL of 1:15 (or 1:10 for two samples) diluted Gram-positive bacterium *Serinicoccus marinus* [29] (process control [22]) was inserted into a 2-mL tube with 1.30 g of 0.2-mm steel beads (Next Advance Inc., Troy, NY, USA). The tube was then inserted into the Terralyzer. The bead-beating protocol for all samples was 2 × 10 s with a 20 s break (apart from samples GTT33 and KxTC22, for which it was 2 × 20 s with a 10 s break).

### 2.6. OralDisk Design and Workflow for Fully Automated Real-Time PCR

**Figure 2:**
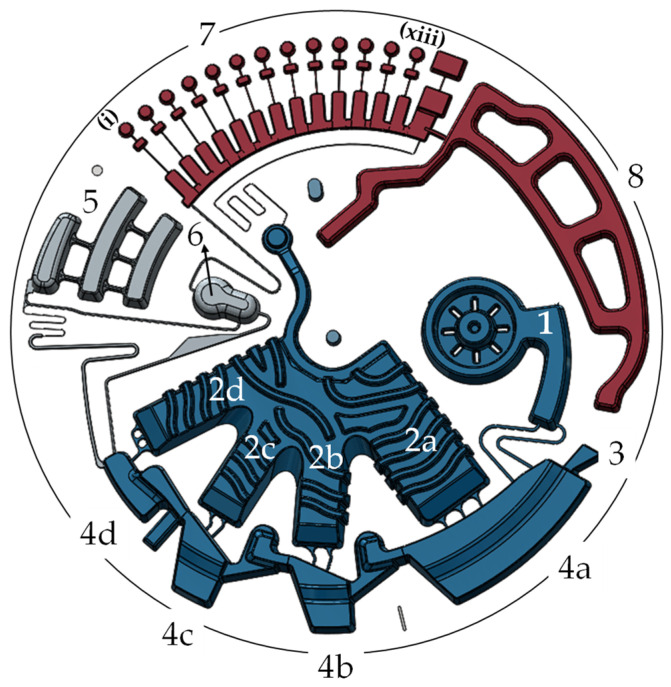
OralDisk design. Blue sector: magnetic bead-based extraction and purification of DNA. #1: sample inlet; #2: stickpacks for storage of buffers for: binding (2a), 1st washing (2b), 2nd washing (2c) and elution (2d); #3: pre-stored (air-dried) magnetic beads; #4: chambers for binding (4a), washing (4b, 4c) and elution of DNA from magnetic beads (4d). Grey sector: eluate transfer module, automating the inward pumping (#5) and eluate mixing with the lyopellet (#6). Red sector: amplification module, automating the preparation and execution of the real-time PCR in structure #7 in the reaction chambers labelled as (i)–(xiii). Structure #8 assists the liquid transfer from chamber #6 to the PCR structure #7.

Oral bacteria were detected using the centrifugal microfluidic OralDisk, which incorporated all the microfluidic unit operations [30] required for the fully automated analysis of whole saliva samples. A volume of 400 µL of ex situ homogenized saliva (Section 2.5) was inserted into the OralDisk (Figure 2, #1) and on-disk DNA extraction and purification was based on a bind-wash-elute protocol [31]. Dedicated buffers were developed by magtivio B.V., the Netherlands, and were stored in pouches (stickpacks [32]) on the disk (Figure 2, #2a–2d). Upon centrifugation (and assisted by controlled heating), liquids were released into their respective (radially outward) chambers (Figure 2, #4a–4d). The stickpacks contained 440 µL of binding buffer (#2a); 200 µL of wash buffer 1 (#2b); 200 µL of wash buffer 2 (#2c); and 180 µL of elution buffer (#2d). Magnetic beads (MagSi-DNA mf beads, ferrimagnetic core with silica shell, cat. no. MD0200010002) were developed by magtivio B.V. especially for microfluidic use for this application and were dry-stored on the disk (Figure 2, #3). Upon magnetic bead rehydration by the binding buffer and lysate, the magnetic beads captured the DNA and were transported through the subsequent chambers (Figure 2, #4a–4d) by means of controlled continuous disk rotation and integrated magnets [33]. In chamber #4d (Figure 2), the DNA was eluted from the magnetic beads, and 160 µL of the eluate was pumped radially inwards into chamber #6, through structure #5 and by means of temperature change-rate (TCR) actuated valving [34] and centrifugo-dynamic inward pumping [35]. In chamber #6, the amplification reagents were pre-stored in the form of a lyophilized pellet (46 µL; TaqMan^®^ Lyophilized 1-Step qPCR Master Mix; 3.5×, Thermo Fisher Scientific, USA). Upon lyopellet rehydration and thorough mixing using a dedicated microfluidic protocol to ensure homogeneity [36], the mixture was aliquoted [37] into the PCR reaction chambers (#7) where the primers/probes for each oral bacterium (plus those for the control bacterium *S. marinus*) were dry-stored. Chamber (i) was a sacrificial chamber to collect residual liquid. Upon rehydration, the thermocycling protocol for real-time PCR started: 95 °C for 3 min (initial denaturation) and 40 cycles of 95 °C for 10 s and 60 °C for 30 s. 

The microfluidic protocol comprised a slightly modified version of a protocol previously published by the authors [21]. According to this slightly modified protocol, the times lapsed during the microfluidic processes in the blue-, grey- and red-marked modules in Figure 2 were: ~37 min, ~12 min and ~107 min, respectively (the latter including the PCR thermocycling). Notably, the OralDisk does not have any sample outlet port but is a closed system, as are all microfluidic cartridges in similar systems, so that the amplified DNA does not contaminate the cartridge processing instrument, thereby risking false positive results during the next measurement. Since we used human sample material, the disposal of the OralDisk was performed by autoclaving, as for other typical laboratory consumables (e.g., wells, tubes) that are used in nucleic acid amplification practices. 

**Figure 3:**
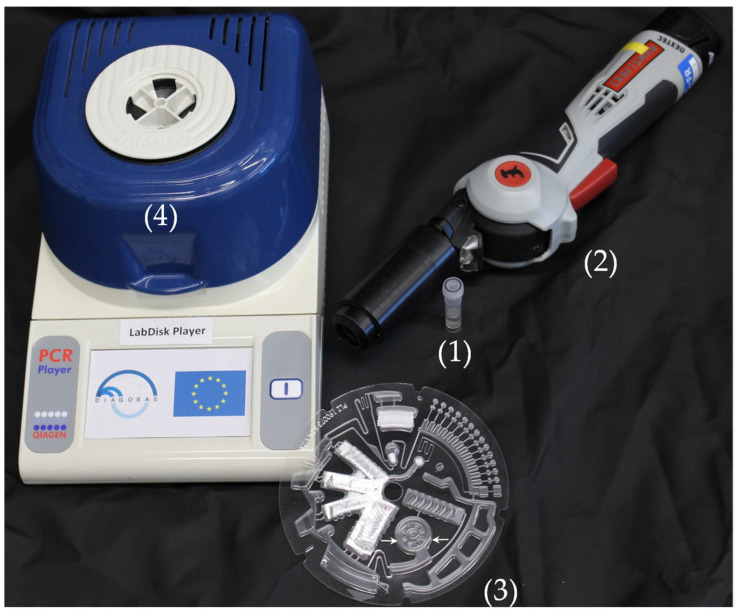
Image of the items which comprise the experimental setup. (**1**) Tube containing the mixture of the saliva sample, *S. marinus* control bacterium and steel beads. (**2**) Hand-held device (Terralyzer), into which the tube is inserted for performing the mechanical lysis and saliva homogenization. (**3**) The OralDisk, where the lysate is pipetted in the chamber indicated by the two white arrows. (**4**) The LabDisk Player instrument that performs the OralDisk processing and real-time PCR.

PCR thermocycling was performed in a customized LabDisk processing device functional model (QIAGEN Lake Constance, currently DIALUNOX GmbH, Lake Constance, Germany) (Figure 3) comprising: (i) a thermal module that enables global air heating for performing the necessary thermocycling protocols; (ii) a mechanical module for the precise positioning, acceleration and deceleration of the disks; (iii) an optical module for detection of the real-time fluorescence signal derived from the nucleic acid amplification product; and (iv) integrated magnets for bead transfer during the DNA extraction and purification. The raw data acquired with the LabDisk Player were analyzed using a RotorGene (QIAGEN, Hilden, Germany) software program to acquire Cq values.

### 2.7. OralDisk Fabrication

The OralDisks were fabricated by microthermoforming [38,39] of polycarbonate (PC) polymer foils (250 µm thickness, Makrofol^®^ DE 1-1 000000, Covestro, Leverkusen, Germany) using a hot embossing machine (HEX01, Jenoptik AG, Jena, Germany) at the Hahn-Schickard Lab-on-a-Chip Foundry Service [40]. Microthermoforming technology is well-known from macro-scale blister package fabrication, which has been adapted and transferred to the micro-scale. In short, an elastomeric mold made of poly(dimethylsiloxane) (PDMS) was heated. The overlying polymer foil was heated as well, and at a specific temperature above the foil’s glass transition, air was blown onto it so that it assumed the shape of the mold. Ultimately, this technology possesses the following advantages: (i) it allows monolithic fabrication of the cartridge and (ii) it is scalable and able to produce several tens of thousands of pieces when required. After the foil structuring, a Teflon coating (0.5% *w*/*w* Teflon (Teflon Amorphous Fluoropolymer, Chemours International Operations Sarl, Geneva, Switzerland) in Fluorinert™ FC-770 (art. # F3556-100ML, Sigma-Aldrich Chemie GmbH, Darmstadt, Germany)) was applied to chambers #4a–4d (Figure 2) in order to provide hydrophobic microfluidic properties in the nucleic acid extraction module. Then, 20 µL of magnetic beads with 10 µL of 250 mg/mL trehalose were pipetted into the corresponding chamber (Figure 2, #3), and 3.5 µL of each primer/probe reaction mix with 0.5 µL of 1 M trehalose (final concentration 50 mM) were pipetted into each reaction chamber (Figure 2, #7). The Teflon coating and the drying of the magnetic beads and primers/probes followed a previously published protocol (1 h at 50 °C) [20,41,42]. The lyopellets containing the amplification reagents were manually inserted into the disk. All buffers were stored in dedicated aluminum pouches (stickpacks). The sealing temperature and pressure used to prepare the stickpacks allowed their opening at a specific rotational frequency (70 Hz), whereby buffers were released into the corresponding chambers of the extraction module (Figure 2). The stickpacks were also manually inserted into the disk. The whole cartridge was sealed using a pressure-sensitive adhesive foil (9795R, 3M, Maplewood, MN, USA). The cartridge was then inserted into an aluminum pouch with a nitrogen atmosphere and desiccant bags, and was stored at room temperature until use.

### 2.8. Statistics

The *p*-values for *T. forsythia* were calculated using the pairwise Wilcoxon Rank Sum Test (statistical software R [43] including the package *tidyverse* [44]) without correction for multiple testing and with a significance level of 0.05. *p*-values were also calculated for *P. gingivalis* but were higher than 0.05 with both the OralDisk and the lab-based reference method. For these two bacteria, all Cq measurements were available (i.e., no ‘ND’ values in Supplementary Table S1). For the other eight bacterial species, one or more Cq values were missing and were deemed to be beyond the limit of detection (marked as ‘ND’ in Supplementary Table S1). These datasets were not used for the calculation of *p*-values. For all species and diagnosis groups, we generated boxplots (Supplementary Figure S1) using Origin^®^2019 software (version 2019 (9.60)) and included a calculation of the median values and interquartile ranges (IQR) of the Cq results (Supplementary Table S2).

## 3. Results and Discussion

### 3.1. Real-Time PCR on the OralDisk

**Figure 4:**
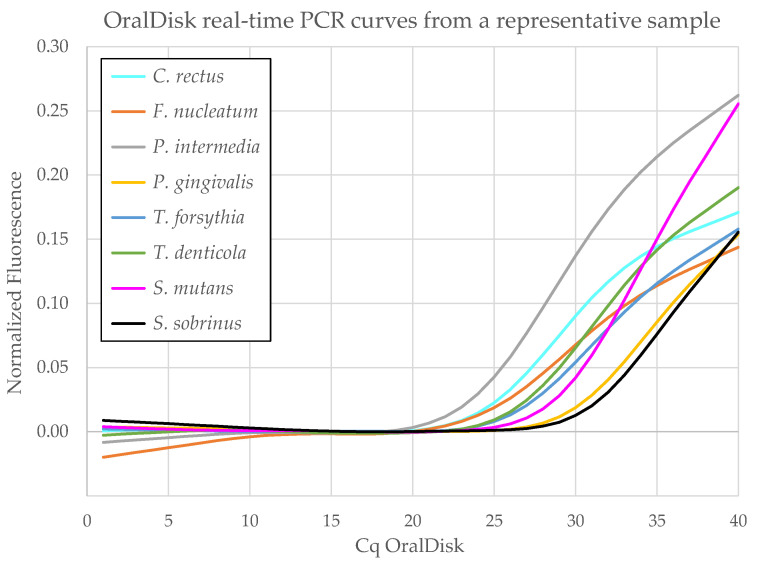
Representative real-time PCR curves for the oral bacteria detected with the OralDisk in one whole saliva sample.

In this study, 25 disks were used to test 24 clinical samples (from seven healthy, nine caries and eight periodontitis patients) and one negative control (H_2_O). Each sample was tested once per disk. Figure 4 shows representative OralDisk real-time PCR curves from which the Cq values were calculated for all bacteria that were detected in a single sample. The Cq values of all bacteria in all the samples tested with OralDisks are summarized in Supplementary Table S1. These values were used for the subsequent analysis and the comparison with the lab-based reference and commercial iai PadoTest methods. The methods were compared by means of: (i) the number of assay targets detected as positive and negative by the OralDisk, the iai PadoTest and the lab-based reference method and (ii) the scatter plots of the acquired Cq values for each clinical diagnosis group and for each bacterium for the OralDisk and the lab-based reference method. These analyses were performed in order to examine possible trends in the data.

### 3.2. Performance Comparison between the OralDisk and the Lab-Based Reference Method

Each saliva sample was tested using one OralDisk, which simultaneously screened for ten bacterial species by means of its geometric multiplexing configuration. None of the samples tested were found to contain all ten of the bacterial species screened for. This was in line with our previous findings using a full cohort study, in which the corresponding lab-based PCR reference method was used [22]. In order to assess the degree of qualitative agreement (i.e., bacterial presence/absence) between the OralDisk and the corresponding lab-based reference method, we divided the results (Table 1) into the following four groups:

(a) assay targets detected as positive by both the OralDisk and the lab-based reference (agreement in positive samples: 154/175 (88.0%) cases);
(b) assay targets detected as positive by the OralDisk but negative by the lab-based reference (disagreement in 7/59 (11.9%) cases);
(c) assay targets detected as negative by both the OralDisk and the lab-based reference (agreement in negative samples: 52/59 (88.1%) cases);
(d) assay targets detected as negative by the OralDisk but positive by the lab-based reference (disagreement in 21/175 (12.0%) cases).

A comparison between the OralDisk and the lab-based reference results was performed (i) for each clinical diagnosis group and (ii) for each bacterial species. Regarding the former, the highest agreement between the two methods in terms of positively detected targets (group (a)) was found in the caries samples (91.9%), followed by the healthy samples (85.4%) and, finally, the periodontitis samples (85.0%). Regarding the latter, the positive agreement between the two methods ranged from 85.7% to 100% for *C. rectus*, *P. intermedia*, *P. gingivalis*, *T. denticola*, *S. mutans* and *S. sobrinus*. Lower agreement between the OralDisk and the lab-based reference positives was observed for *F. nucleatum* (16/23 (69.6%) cases) and *Lactobacillus* spp. (7/11 (63.6%) cases). 

It is important to mention that each sample was analyzed with three technical replicates with the lab-based PCR reference compared to one with the OralDisk to simulate the POC workflow. In three cases within group (d), the lab-based PCR reference did not give identical results for the triplicates. In two cases, two of three repeats for *F. nucleatum* were detected positive and in one case, one of three repeats for *Lactobacillus* spp. was detected positive. This may imply that these particular PCR assays in those samples were close to the limit of detection for the lab-based reference, which would explain why they were missed by the OralDisk. Finally, the OralDisk detected *A. actinomycetemcomitans* in only one of the four lab-based reference positive samples. In line with data from the preceding clinical study, this species was not often detected among the samples, and when detected, it was often associated with very low levels of target genome equivalents [22]. This species also did not appear to play any discriminatory role between the healthy, caries and periodontitis groups in the aforementioned study and for the recruited age groups [22]. However, *A. actinomycetemcomitans* was included in the panel because it may play a role in cases where early onset periodontitis (i.e., younger patient ages than usual) was suspected.

A possible source of disagreement between the two methods may be the different approaches for bacterial lysis, DNA extraction and purification prior to PCR amplification. The lab-based reference method used an enzymatic lysis methodology (lysozyme, mutanolysin, proteinase K enzymes [22]) with prolonged incubation times, followed by column-based purification. For the OralDisk, mechanical lysis was performed ex situ using a hand-held bead-beating device (Terralyzer, Zymo Research, USA), followed by a magnetic bead-based bind-wash-elute protocol [31] for DNA extraction and purification on the disk. Enzymatic or mechanical lysis may be more efficient, depending on the cell wall properties of certain bacterial species—the OralDisk panel included both Gram-positive and Gram-negative bacteria (Section 2.2). 

Discrepancies in DNA extraction efficiency may also be expected between the different approaches (column versus bead-based), as well as between different test devices. These factors may have an impact on the detection of low abundances of bacteria (i.e., higher Cq values). Consequently, targets that are close to the limit of detection of the OralDisk (in its current configuration) may still be detectable by the lab-based reference method. This could partly account for discrepancies between the two datasets where the lab-based reference, but not the OralDisk, appeared to detect certain bacterial species more frequently.

### 3.3. Performance Comparison between the OralDisk and the Commercial iai PadoTest

The iai PadoTest (iai PadoTest, Institut für Angewandte Immunologie IAI AG, Zuchwil, Switzerland), a commercially available system for the detection of periodontal pathogens, was used to analyze 18 out of the 24 samples. We compared the data obtained by the iai PadoTest and the OralDisk for these 18 samples and for five species per sample (as not all ten species were shared between these two methods). To enable a direct interpretation of the results, we only compared the qualitative outcomes of the methods (i.e., presence/absence of the target bacteria), as the OralDisk in this study did not provide quantitative values for bacteria concentrations. The results from the OralDisk and iai PadoTest are summarized in Table 2. The iai PadoTest positively detected 28 of the microbial target assays, while the OralDisk detected the same 28 and 33 more, thus giving 61 in total. Possible explanations for this discrepancy in detection between the two methods may be the different molecular identification principles and/or assay protocols used [22,27]. In fact, the iai PadoTest is designed to examine gingival crevicular fluid (GCF). However, for better comparability with the lab-based reference and the OralDisk we used a modified protocol based on saliva, as described and discussed previously [22].

### 3.4. Comparison of Cq Performance 

**Figure 5:**
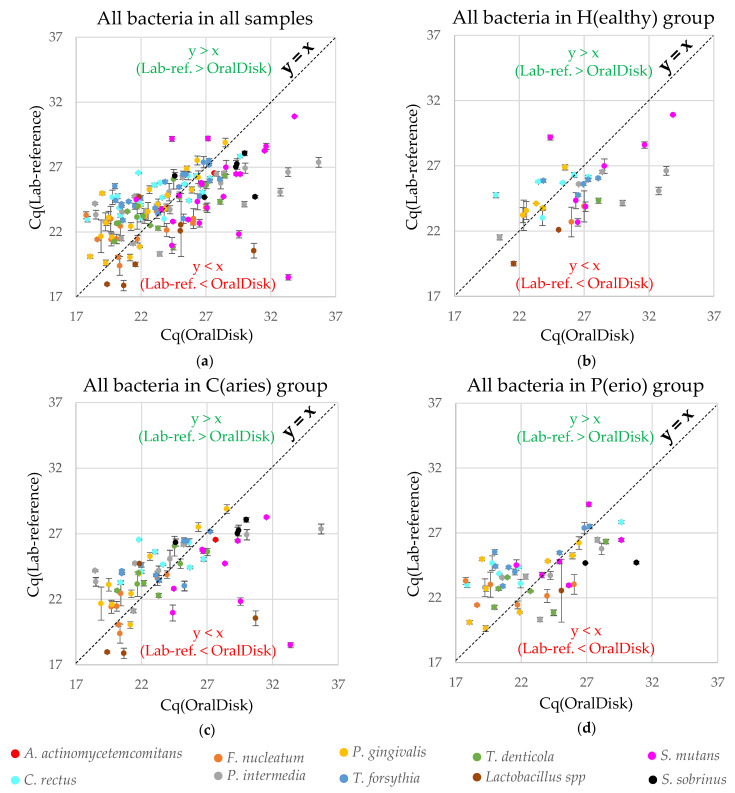
Scatter plots correlating the Cq values from the OralDisk and the lab-based reference for all bacteria: (**a**) in all three groups; (**b**) in the healthy group; (**c**) in the caries group; and (**d**) in the periodontitis group. Similar scatter plots for each individual bacterium are depicted in the Supplementary Figure S2.

The performance comparison between the OralDisk and the lab-based reference (Table 1) does not consider the Cq values of the two methods. In this section, the Cq values of group (a) (Section 3.2) are therefore shown as scatter plots, in order to observe whether one of the two methods exhibited any trend in Cq for all bacteria in all samples, as well as for all bacteria in each clinical diagnosis group (Figure 5). Each data point corresponds to the detection of a specific bacterial species in a specific sample with both methods. The *y*-axis error bars are derived from the standard deviations of triplicate (or in some cases duplicate) measurements made with the lab-based reference PCR method [22]. The calculation of such standard deviations was not possible for the *x*-axis (OralDisk PCR), as each sample was tested with only one OralDisk cartridge.

The diagonal line y = x is shown in the scatter plots simply to assist the observation and assessment of whether the OralDisk or the lab-based reference method show any trend in specific Cq areas. Data points above the y = x line mean that for a specific measurement, Cq(Lab-reference) > Cq(OralDisk), while data points below the y = x line mean that Cq(OralDisk) > Cq(Lab-reference). Interestingly, it appears that for the healthy group, more data points lie below the y = x line, while for the caries and periodontitis groups and for all samples combined, the number of points above and below the x = y line appear to be balanced (Table 3). When deriving a linear fit trendline, the slope of Cq(Lab-reference)/Cq(OralDisk) is very similar between the total, healthy, caries and periodontitis groups (0.34, 0.38, 0.32, 0.36, respectively). This is an indication that the relation between the two methods is consistent and independent of the nature of the three groups included in the study. From qualitative observation, there seems to be a center of the scatterplot clusters at Cq ~ 24. Across the area where the Cq is higher than ~24 (i.e., at lower bacterial concentrations), the OralDisk method tends to generate positive signals at higher Cq values than the lab-based reference. This further supports the hypothesis expressed in Section 3.2, namely that lower bacterial concentrations that can still be detected by the lab-based reference may be missed by the OralDisk. Conversely, across the area where Cq values were lower than ~24 (i.e., at higher bacterial concentrations), increasing PCR signals tend to be observed earlier for the OralDisk.

As each OralDisk analyzes the presence/absence of ten bacteria simultaneously, the scatter plots in Figure 5 represent the entire panel and contain the collective information from all bacteria. In order to investigate whether some individual bacteria exhibit any specific trends, we composed the scatter plots for each bacterium for all clinical diagnosis groups. From Supplementary Figure S2, we observe different tendencies for some bacteria (quantitatively summarized in Table 3). For example: (i) *C. rectus*, *P. gingivalis* and *T. forsythia* tend to appear in the Cq(Lab-reference) > Cq(OralDisk) area (i.e., the OralDisk amplification curves yielded earlier Cq values than the lab-based reference); (ii) *S. mutans* tends to appear in the Cq(Lab-reference) < Cq(OralDisk) area (i.e., the OralDisk amplification curves yielded later Cq values than the lab-based reference); and (iii) there is no trend for *F. nucleatum*, *P. intermedia* and *T. denticola*.

We further analyzed the Cq values from the OralDisk and the lab-based reference and mapped their distributions per bacterial species for all three clinically diagnosed groups (Supplementary Figure S1). It should be noted that due to the small number of measured samples, clinical conclusions cannot be extracted from the results per se. From the descriptive graphical representation, it can be observed that for *P. intermedia*, *P. gingivalis* and *T. forsythia* (and possibly also *T. denticola*, although N = 2 for the OralDisk), the boxplots would tend to be distinguishable (especially if/when a higher number of samples were tested), even though there are overlapping standard deviations. In cases where testing a higher number of samples would lead to a smaller standard deviation, these four bacteria would possibly be the first ones that the OralDisk would detect as differentiators between clinical diagnosis groups. Within this small-scale study, the tendency towards differentiation seems to be stronger with the OralDisk than with the lab-based reference, as the median lines of the OralDisk results for the different diagnosis groups seem to be further apart (even though not necessarily lower) than those of the lab-based reference, especially between the healthy and the caries groups (actual median values given in Supplementary Table S2). Indicative of this (and being aware of the small number of samples for thorough statistical analysis), for *T. forsythia*, the *p*-value between the healthy and the caries groups was 0.033 with the OralDisk and 0.29 with the lab-based reference, and between the healthy and the periodontitis groups, it was 0.057 with the OralDisk and 0.29 with the lab-based reference (Section 2.8).

### 3.5. Overall Evaluation of the OralDisk

The development and implementation of chair-side molecular diagnostics in the field of oral health lags behind many other fields of healthcare, including infectious diseases such as respiratory tract, bloodstream and gastrointestinal infections, for which point-of-care or near-patient systems are commercially available or at the product development stage [45]. However, the socioeconomic burden of oral diseases is high, with expenditure for the treatment of dental diseases reaching EUR 90 billion and additional productivity losses of over EUR 50 billion in EU member states in 2015 [46]. Furthermore, the documented relationship between periodontal and systemic diseases [8,47,48,49,50] has started to raise awareness of the importance of oral health and especially of early diagnosis, prevention and post-treatment monitoring. 

Table 4 summarizes some existing technologies, together with the bacterial panels they detect. A commercial test for the biomarker-based detection of periodontitis is available from Dentognostics GmbH (PerioSafe^®^) [51]. It detects a single protein marker, namely the active matrix metalloproteinase-8 (aMMP-8) [52,53,54] but no bacteria per se. Furthermore, although the molecular-based detection of oral bacteria has been reported by MyPerioPath^®^ [55], HR5^TM^ (High Risk Pathogen Test from Direct Diagnostics [56]) and iai PadoTest [27], all these methods are not chair-side-compatible but laboratory-based. Thus, the samples need to be transported to a laboratory and the results are only available after some days, whereas the OralDisk delivers results in <3 h. The PerioSafe^®^ and PerioPOC^®^ [57,58] require only 5 and 20 min, respectively, due to their lateral flow configuration. However, the panels of both tests are limited: for the former, to a single protein biomarker; for the latter, to five periodontitis pathogens. Additionally, none of the methods in Table 4 detects caries-related bacteria but focus only on periodontitis-associated bacteria. Interestingly, in terms of throughput, all chair-side technologies test one sample per run. For the lab-based technologies, the number of samples tested per run may depend on the logistics of the particular laboratory. The overarching features of the OralDisk platform compared to the listed systems are that it detects ten major caries- and periodontitis-related bacteria simultaneously from non-invasively collected whole saliva using molecular-based detection and a chair-side compatible system.

In terms of specimens, some of the methods listed in Table 4 use paper points from periodontal probing (iai PadoTest, PerioPOC^®^). However, paper points have the inherent disadvantage that they are invasive and only reflect the local bacterial distributions of specific tooth pockets [12,13]. Saliva is an increasingly popular candidate for analysis as it is easy to collect in tubes even in milliliter volumes and its collection is non-invasive in nature [59,60]. The OralDisk requires 400 µL of saliva (compared to 3–4 drops of oral rinse for PerioSafe^®^ and 160 µL of lysis solution for the immersion of paper points of PerioPOC^®^, with 20 µL of this volume being added to the lateral flow chip). For the lab-based methods of iai PadoTest, MyPerioPath^®^ and HR5^TM^ the required volume is not known. In any case, the availability of large amounts of saliva makes the required volume of this specimen type less critical in terms of the test to be selected. Non-oral-related diagnostics have also attempted to use saliva to detect biomarkers or nucleic acids in diseases such as COVID-19, type 2 diabetes mellitus, cardiovascular diseases and Alzheimer’s disease [61,62,63,64,65,66,67]. The specimens used in the current study were whole saliva samples derived from recruited individuals of diverse oral health background (healthy, caries, periodontitis) during a clinical study [22]. Compared to spiked samples with known compositions, this methodology offers decisive advantages: (i) the oral flora in healthy and diseased individuals is best represented in saliva, since it already contains many of the bacteria to be examined and (ii) for POC use, it is essential to ensure that the OralDisk is compatible with the physical, biological and chemical properties of the natural matrix, i.e., whole saliva and not any artificial saliva matrix. Indeed, the compatibility of the LabDisk with this complex matrix paves the way for applicability of the platform in areas that can make use of this abundant and easy-to-acquire specimen.

An outstanding advantage of the OralDisk is the automated sample processing and detection of the assay targets. The system is based on a microfluidic platform that integrates all the necessary biochemical reagents and operations in a protocol requiring minimal and simple hands-on work. The only short manual step is the homogenization of whole saliva using a bead-beating hand-held device (Terralyzer, Zymo Research, USA), which combines homogenization and bacterial lysis in one step (saliva bead-beating has also been previously reported as a lysis method for lab-based downstream analysis [69,70]). Even in such a configuration, the hands-on work was estimated to be only 10 min, including the ex situ saliva homogenization and mixing with the control bacterium *S. marinus*, the pipetting into the disk, and the insertion of the latter into the processing instrument. This time is far shorter than the manual sample preparation time (at least 1.5 h) that is required prior to a laboratory-based PCR. Additionally, previously published work by the authors has already demonstrated the use of an on-disk microfluidic unit operation for saliva homogenization [68] (as a pre-analytic approach for downstream protein analysis), which uses disk-integrated magnets actuated by magnets above the disk [33]. This disk-compatible approach is planned to be tested in the future as an in situ lysis and homogenization step. It will be integrated with the DNA purification module and will thus replace the only manual step (i.e., bead-beating with the mobile Terralyzer device) of the current OralDisk protocol. Further automation can be achieved if the aforementioned in situ mechanical lysis step is combined with downstream dilution and direct amplification (after biochemical optimization), without the need for a bead-based bind-wash-elute protocol. This modification is expected to reduce the total time-to-result by approximately 0.5 h. In addition, a drastic reduction in the time-to-result is also planned to be achieved by means of a new LabDisk Player that will implement contact heating using Peltier elements instead of air heating. This method is expected to reduce the PCR time from ~2 h to < 0.5 h, thereby resulting in an estimated total time-to-result of <1 h.

The major feature of automation of the OralDisk workflow leads also to a drastic reduction of hands-on work and time that is required by laboratory personnel, which can result in a reduction of the overall ‘hidden costs’ of laboratory workflows. Even though we cannot assess the end-user transfer price of the OralDisk (as it is currently at development stage), the actual cost-driver aspects have been identified, as well as the actions that need to be taken at the product development stage, e.g., re-assignment from thermoforming to injection molding fabrication method (for manufacturing of hundreds of thousands of OralDisk cartridges); screening for more cost-effective polymeric cartridge materials and the complete automation of the entire manufacturing workflow. In any case, the price of such a diagnostic system should not be compared with single-biomarker or single-parameter detection systems, as the OralDisk offers an increased range of information for a high number of pathogens and for both periodontitis and caries diseases. In fact, the realistic goal for the OralDisk is that the cost per pathogen tested becomes significantly lower than that using a laboratory test.

The analytics were performed on a batch of samples sufficiently representative to allow the platform to demonstrate its performance and applicability for the detection of a broad panel of ten Gram-negative and Gram-positive bacterial species. As previously mentioned, this means that both periodontitis and caries can be monitored simultaneously on a single OralDisk. Periodontitis has been mainly associated with Gram-negative anaerobic bacteria, while caries has been mainly associated with Gram-positive carbohydrate-fermenting bacteria [71]. The OralDisk contains one of the broadest panels available for bacterial detection among comparable systems (Table 4). This is achievable due to the multiple reaction chambers (#7, Figure 2) that enable geometric multiplexing. The level of multiplexing can be further increased by multiple wavelength detection in the same chamber (color multiplexing). For example, in this publication the Gram-positive bacterium *S. marinus* was included as a process control and underwent the same lysis, extraction, purification and amplification processes as the oral bacteria present in the test sample. *S. marinus* primers/probes were included together with the bacteria-specific primers/probes in each reaction chamber, providing an indication that the internal biochemical and microfluidic processes had been correctly implemented. In the disks that we tested, there was no case where the *S. marinus* was not detected, thereby confirming qualitatively that the sample-to-answer process functioned successfully. The simultaneous detection of several bacteria, including the process control, within a single sample is a major achievement of the platform, which until now had demonstrated its capability for infectious diseases that are associated with either one or two pathogens, such as sepsis [18], tropical infections [20] and respiratory tract infections [19,21].

The ~90% agreement between the OralDisk and the lab-based reference amongst targets detected as positive and negative indicates that the OralDisk platform may be suitable for accurate molecular-based oral bacteria detection. Any differences could be attributable to the different lysis, extraction and/or test device approaches utilized by the two methods. An increase in the test sensitivity of the OralDisk might be achievable using a pre-amplification step, which in a past application of the LabDisk technology was shown to detect down to a few bacteria/mL [18]. However, high sensitivity may not be particularly crucial for oral health screening purposes. For example, the oral microbiota is diverse in both healthy and diseased patients, including both commensals and opportunistic pathogens. It is the dynamic changes over time of bacteria among the oral microbiota, rather than their mere presence/absence, that drives the dysbiotic changes that lead to oral disease [72]. The fact that the OralDisk has demonstrated its capacity to analyze oral bacteria provides a basis upon which the platform can be implemented in time-course studies associated with patient monitoring, similar to the rationale reported by Paqué et al. [73]. In such future studies, we will have the opportunity to proceed to the quantification of bacteria, using OralDisk-derived calibration curves to convert Cq values into numeric bacterial loads.

The LabDisk and the corresponding customized processing device used in this work are amenable to further performance improvements (such as inclusion of the lysis step on the disk and the subsequent reduction of the time-to-result) and may be applicable as an auxiliary tool for oral microbial screening in dental POC settings. Finally, for truly holistic monitoring of oral health, bacteria-based microbiological examination in the OralDisk will be combined with protein biomarker concentration monitoring. The LabDisk has already been shown to be compatible with immunoassays by the running of a basic reaction (without detection) as a proof-of-principle demonstration [74]. A newly developed and tested bead-based immunoassay with oral biomarkers [75] will enable an immunoassay disk to be run in the same instrument as the PCR disk. This will increase the interoperability between immunological and microbiological diagnostic outputs using this platform [76]. Subsequently, by using combined computational technologies and the OralDisk device, new diagnostic predictive models of disease development and progression may be generated [73]. Such developments will enable evidence-based pre- and post-treatment monitoring of a range of oral (OralDisk) and non-oral (application-specific LabDisk) diseases.

## 4. Conclusions

This technical feasibility study demonstrated the ability of the OralDisk to detect a broad range of seven periodontitis- and three caries-related bacteria from whole saliva samples in < 3 h, using an automated platform for molecular-based detection. Importantly, the platform was proven to be compatible with saliva as a sample matrix for the first time, which paves the way for (i) ‘modernization’ of contemporary oral health by using a molecular-based diagnostic tool for saliva (instead of probe-based) close to the chair-side practice, acting supportively to ‘traditional’ methods and (ii) implementation in further areas of non-invasive saliva-based diagnostics. Compared to the lab-based reference test, the OralDisk results showed ~90% agreement amongst targets detected as positive and negative. We observed that higher levels of bacteria (Cq values < 24) generally resulted in lower Cq values with the OralDisk compared to the lab-based reference method. This was primarily observed for *C. rectus, P. gingivalis* and *T. forsythia*. On the other hand, lower bacterial levels (Cq values > 24) were mostly detected later with the OralDisk compared to the lab-based reference method. This was primarily observed for *S. mutans*.

Optimization of the platform towards automation and reduction of the time-to-result will further increase its potential for adoption at the chair-side. The future goals are to apply the OralDisk platform in larger clinical studies and to determine its clinical potential in (i) providing early detection/prevention of oral diseases before they are detectable with a conventional clinical examination or radiographic methods and (ii) monitoring progress during and after periodontal treatment by providing quantitative information on changes in bacterial load. Furthermore, such a diagnostic system could potentially contribute to more informed decision making regarding the prescription of oral antibiotics, while also acting as an early warning system for underlying systemic diseases [77] such as diabetes and cardiovascular diseases, which have previously been correlated with periodontitis.

## Figures and Tables

**Table 1:** Comparison of the lab-based reference versus the OralDisk for positive/negative results. Each number in the table denotes the number of times an assay target was detected in the OralDisk and lab-based reference experiments. ‘Ch.1–Ch10’ refers to the numbering of the respective OralDisk chamber where each assay target was detected. ‘Pos.’ and ‘Neg.’ are abbreviations for ‘positive’ and ‘negative’, respectively.

	Ch.1	Ch.2	Ch.3	Ch.4	Ch.5	Ch.6	Ch.7	Ch.8	Ch.9	Ch.10	
	*A. actinomycetemcomitans*	*C. rectus*	*F. nucleatum*	*P. intermedia*	*P. gingivalis*	*T. forsythia*	*T. denticola*	*Lactobacillus* spp.	*S. mutans*	*S. sobrinus*	Number of Pos., Neg. over Total Assay Targets
Lab-reference Pos.	4	23 ^a^	23 ^a^	24	22 ^a^	22 ^a^	15	11	24	7	175/234
Lab-reference Neg.	20	0	0	0	0	0	9	13	0	17	59/234
(a) OralDisk Pos. and Lab-reference Pos.	1	21	16	22	22	22	15	7	22	6	154/175 (88.0%)
(b) OralDisk Pos. and Lab-reference Neg.	0	0	0	0	0	0	2	5	0	0	7/59 (11.9%)
(c) OralDisk Neg. and Lab-reference Neg.	20	0	0	0	0	0	7	8	0	17	52/59 (88.1%)
(d) OralDisk Neg. and Lab-reference Pos.	3	2	7	2	0	0	0	4	2	1	21/175 (12.0%)

^a^ In one disk, signals of *C. rectus* and *F. nucleatum* where not measured, while in two (other) disks, signals of *P. gingivalis* and *T. forsythia* were not measured. For reasons of comparison, these assays were excluded from the lab-based reference.

**Table 2:** Comparison between the iai PadoTest and the OralDisk. ‘Ch.1–Ch10’ refers to the numbering of the OralDisk chambers where each bacterium was detected. Grey shading denotes the bacteria that are not included in the iai PadoTest panel. ‘Pos.’ and ‘Neg.’ are abbreviations for ‘positive’ and ‘negative’, respectively.

	Ch.1	Ch.2	Ch.3	Ch.4	Ch.5	Ch.6	Ch.7	Ch.8	Ch.9	Ch.10	
	*A. actinomycetemcomitans*	*C. rectus*	*F. nucleatum*	*P. intermedia*	*P. gingivalis*	*T. forsythia*	*T. denticola*	*Lactoba-* *cillus* spp.	*S. mutans*	*S. sobrinus*	Number of Pos., Neg. Assay Targets
OralDisk Pos.	1			16	16	16	12				61
iai PadoTest Pos.	0			7	8	3	10				28
OralDisk Neg.	17			2	0	0	6				25
iai PadoTest Neg.	18			11	8	13	8				58

**Table 3:** Number of cases where the Cq(Lab-reference) was higher or lower than the Cq(OralDisk). Calculations are based on the scatter plots in Figure 5. The bacteria *A. actinomycetemcomitans*, *Lactobacillus* spp. and *S. sobrinus* are given only indicatively as their total cases were very few.

	Number of Cases with Cq(Lab-Reference) > Cq(OralDisk)		Number of Cases with Cq(Lab-Reference) < Cq(OralDisk)		TOTAL Cases
For all bacteria in all groups	80	51.9%	74	48.1%	154
For all bacteria in healthy group	12	35.3%	22	64.7%	34
For all bacteria in caries group	41	55.9%	30	44.1%	68
For all bacteria in periodontitis group	27	57.7%	22	42.3%	52
For *A. actinomycetemcomitans* in all groups	0	0.0%	1	100.0%	1
For *C. rectus* in all groups	15	71.4%	6	28.6%	21
For *F. nucleatum* in all groups	8	53.3%	7	46.7%	15
For *P. intermedia* in all groups	10	45.5%	12	54.5%	22
For *P. gingivalis* in all groups	17	77.3%	5	22.7%	22
For *T. forsythia* in all groups	16	72.7%	6	27.3%	22
For *T. denitcola* in all groups	8	50.0%	8	50.0%	16
For *Lactobacillus* spp. in all groups	1	14.3%	6	85.7%	7
For *S. mutans* in all groups	4	18.2%	18	81.8%	22
For *S. sobrinus* in all groups	1	16.7%	5	83.3%	6

**Table 4:** List of basic performance characteristics for a range of molecular-based methods of oral health assessment. ‘LFT’ stands for ‘Lateral Flow Test’. Bacteria that are not included in the panels of some technologies are marked with ‘X’.

Characteristics	PerioSafe^®^	iai PadoTest	MyPerioPath^®^	PerioPOC^®^	HR5^TM^	OralDisk
*Saliva sampling*	Yes (Oral rinse)	No (Probe)	Yes	No (Probe)	Yes	Yes
*Chair-side compatible*	Yes	No (Requires lab)	No (Requires lab)	Yes	No (Requires lab)	Yes ^a^
*Quantitative capacity*	Yes/No (LFT) ^b^	Yes (qPCR)	Yes (qPCR)	No (LFT) ^c^	Yes (qPCR)	Yes (qPCR)
*Caries panel included*	No	No	No	No	No	Yes
*Number of bacteria in panel, tested per run*	None (detects aMMP-8 biomarker)	6	11	5	5	10
**Bacteria panel**						
*Aggregatibacter actinomycetemcomitans*	-	X	X	X	X	X
*Tannerella forsythia*	-	X	X	X	X	X
*Porphyromonas gingivalis*	-	X	X	X	X	X
*Treponema denticola*	-	X	X	X	-	X
*Prevotella intermedia*	-	X	X	X	X	X
*Filifactor alocis*	-	X	-	-	-	-
*Fusobacterium nucleatum*	-	-	X	-	X	X
*Campylobacter rectus*	-	-	X	-	-	X
*Capnocytophaga* species (gingivalis, ochracea, sputigena)	-	-	X	-	-	-
*Streptococcus mutans*	-	-	-	-	-	X
*Streptococcus sobrinus*	-	-	-	-	-	X
*Lactobacillus* spp.	-	-	-	-	-	X
*Peptostreptococcus micros*	-	-	X	-	-	-
*Eikenella corrodens*	-	-	X	-	-	-
*Eubacterium nodatum*	-	-	X	-	-	-

^a^ Once the mechanical lysis of bacteria is transferred from the Terralyzer into the OralDisk [68]. ^b^ Lateral Flow Test (protein detection) [50,51,52,53]. Visual readout provides qualitative analysis. Quantitative analysis requires a processing instrument. ^c^ Lateral Flow Test (nucleic acid hybridization). Visual readout provides qualitative analysis [58].

## Data Availability

Data is contained within the article or in the supplementary material.

## Supplementary Materials

The following are available online at https://www.mdpi.com/article/10.3390/bios11110423/s1, Table S1: Raw experimental data (Cq values) from the OralDisk and the lab-based reference method, as well as qualitative data from the iai PadoTest. Figure S1: Boxplots of the Cq values for each bacterium detected by the OralDisk and the lab-based reference. Table S2: Median and interquartile range (IQR, describing the middle 50% of values when ordered from lowest to highest) of the Cq values from the OralDisk and lab-based reference measurements. Figure S2: Scatter plots correlating the Cq values from the lab-based reference and the OralDisk for all bacteria in the healthy, caries and periodontitis groups.

## References

[1] Dental Diseases and Oral Health. www.who.int/oral_health/publications/en/orh_fact_sheet.pdf.

[2] Listl S., Grytten J.I., Birch S. (2019). What is health economics?. Community Dent. Health.

[3] Belibasakis G.N., Lund B., Krüger Weiner C., Johannsen B., Baumgartner B., Manoil C., Hultin M., Mitsakakis K. (2020). Healthcare Challenges and Solutions in Dental Practice: Assessing Oral Antibiotic Resistances by Contemporary Point-of-Care Solutions. Antibiotics.

[4] Suda K.J., Calip G.S., Zhou J.F., Rowan S., Gross A.E., Hershow R.C., Perez R.I., McGregor J.C., Evans C.T. (2019). Assessment of the appropriateness of antibiotic prescriptions for infection prophylaxis before dental procedures, 2011 to 2015. JAMA Netw. Open.

[5] Bernabe E., Marcenes W., Hernandez C.R., Bailey J., Abreu L.G., Alipour V., Amini S., Arabloo J., Arefi Z., Arora A. (2020). Global, Regional, and National Levels and Trends in Burden of Oral Conditions from 1990 to 2017: A Systematic Analysis for the Global Burden of Disease 2017 Study. J. Dent. Res..

[6] WHO, Regional Office for Europe, Oral Health, Data and Statistics. http://www.euro.who.int/en/health-topics/disease-prevention/oral-health/data-and-statistics.

[7] NIDCR, NIH, Periodontal (Hum) Disease. http://www.nidcr.nih.gov/OralHealth/Topics/GumDiseases/PeriodontalGumDisease.htm.

[8] Seitz M.W., Listl S., Bartols A., Schubert I., Blaschke K., Haux C., Van Der Zande M.M. (2019). Current Knowledge on Correlations Between Highly Prevalent Dental Conditions and Chronic Diseases: An Umbrella Review. Prev. Chronic. Dis..

[9] D’Aiuto F., Gable D., Syed Z., Allen Y., Wanyonyi K.L., White S., Gallagher J.E. (2017). Evidence summary: The relationship between oral diseases and diabetes. Br. Dent. J..

[10] Bui F.Q., Almeida-da-Silva C.L.C., Huynh B., Trinh A., Liu J., Woodward J., Asadi H., Ojcius D.M. (2019). Association between periodontal pathogens and systemic disease. Biomed. J..

[11] Petersen P.E. (2009). Global policy for improvement of oral health in the 21st century--implications to oral health research of World Health Assembly 2007, World Health Organization. Community Dent. Oral Epidemiol..

[12] Listgarten M.A. (1980). Periodontal probing: What does it mean?. J. Clin. Periodontol..

[13] Hefti A.F. (1997). Periodontal probing. Crit. Rev. Oral. Biol. Med..

[14] Olsen I. (2008). Update on bacteraemia related to dental procedures. Transfus. Apher. Sci..

[15] Daly C.G., Mitchell D.H., Highfield J.E., Grossberg D.E., Stewart D. (2001). Bacteremia due to periodontal probing: A clinical and microbiological investigation. J. Periodontol..

[16] Preshaw P.M. (2015). Detection and diagnosis of periodontal conditions amenable to prevention. BMC Oral Health.

[17] Mitsakakis K., Stumpf F., Strohmeier O., Klein V., Mark D., von Stetten F., Peham J.R., Herz C., Tawakoli P.N., Wegehaupt F. (2016). Chair/bedside diagnosis of oral and respiratory tract infections, and identification of antibiotic resistances for personalised monitoring and treatment. Stud. Health Technol. Inform..

[18] Czilwik G., Messinger T., Strohmeier O., Wadle S., von Stetten F., Paust N., Roth G., Zengerle R., Saarinen P., Niittymaki J. (2015). Rapid and fully automated bacterial pathogen detection on a centrifugal-microfluidic LabDisk using highly sensitive nested PCR with integrated sample preparation. Lab Chip.

[19] Stumpf F., Schwemmer F., Hutzenlaub T., Baumann D., Strohmeier O., Dingemanns G., Simons G., Sager C., Plobner L., von Stetten F. (2016). LabDisk with complete reagent prestorage for sample-to-answer nucleic acid based detection of respiratory pathogens verified with influenza A H3N2 virus. Lab Chip.

[20] Hin S., Lopez-Jimena B., Bakheit M., Klein V., Stack S., Fall C., Sall A., Enan K., Mustafa M., Liz Gillies L. (2021). Fully automated point-of-care differential diagnosis of acute febrile illness. PLoS Negl. Trop. Dis..

[21] Rombach M., Hin S., Specht M., Johannsen B., Lüddecke J., Paust N., Zengerle R., Roux L., Sutcliffe T., Peham J.R. (2020). RespiDisk: A Point-of-Care platform for fully automated detection of respiratory tract infection pathogens in clinical samples. Analyst.

[22] Paqué P.N., Herz C., Jenzer J.S., Wiedemeier D., Attin T., Bostanci N., Belibasakis G.N., Bao K., Körner P., Fritz T. (2020). Microbial analysis of saliva to identify oral diseases using a point-of-care compatible qPCR assay. J. Clin. Med..

[23] Lamont R.J., Hajishengallis G. (2015). Polymicrobial synergy and dysbiosis in inflammatory disease. Trends Mol. Med..

[24] Bostanci N., Bao K., Greenwood D., Silbereisen A., Belibasakis G.N. (2019). Periodontal disease: From the lenses of light microscopy to the specs of proteomics and next-generation sequencing. Adv. Clin. Chem..

[25] Socransky S.S., Haffajee A.D., Cugini M.A., Smith C., Kent Jr R.L. (1998). Microbial complexes in subgingival plaque. J. Clin. Periodontol..

[26] Haffajee A.D., Socransky S.S., Patel M.R., Song X. (2008). Microbial complexes in supragingival plaque. Oral Microbiol. Immunol..

[27] Website of iai PadoTest. https://www.padotest.ch/en.

[28] Belibasakis G.N., Schmidlin P.R., Sahrmann P. (2014). Molecular microbiological evaluation of subgingival biofilm sampling by paper point and curette. APMIS.

[29] Yi H., Schumann P., Sohn K., Chun J. (2004). Serinicoccus marinus gen. nov., sp. nov., a novel actinomycete with L-ornithine and L-serine in the peptidoglycan. Int. J. Syst. Evol. Microbiol..

[30] Strohmeier O., Keller M., Schwemmer F., Zehnle S., Mark D., von Stetten F., Zengerle R., Paust N. (2015). Centrifugal microfluidic platforms: Advanced unit operations and applications. Chem. Soc. Rev..

[31] Boom R., Sol M.M., Jansen C.L., Wertheim-van Dillen P.M., van der Noordaa J. (1990). Rapid and simple method for purification of nucleic acids. J. Clin. Microbiol..

[32] Van Oordt T., Barb Y., Smetana J., Zengerle R., von Stetten F. (2013). Miniature stick-packaging—An industrial technology for pre-storage and release of reagents in lab-on-a-chip systems. Lab Chip.

[33] Hin S., Paust N., Rombach M., Lüddecke J., Specht M., Zengerle R., Mitsakakis M. Minimizing ethanol carry-over in centrifugal microfluidic nucleic acid extraction by advanced bead handling and management of diffusive mass transfer. Proceedings of the 20th International Conference on Solid-State Sensors, Actuators and Microsystems & Eurosensors XXXIII.

[34] Keller M., Czilwik G., Schott J., Schwarz I., Dormanns K., von Stetten F., Zengerle R., Paust N. (2017). Robust temperature change rate actuated valving and switching for highly integrated centrifugal microfluidics. Lab Chip.

[35] Zehnle S., Schwemmer F., Roth G., von Stetten F., Zengerle R., Paust N. (2012). Centrifugo-dynamic inward pumping of liquids on a centrifugal microfluidic platform. Lab Chip.

[36] Hin S., Paust N., Keller M., Rombach M., Strohmeier O., Zengerle R., Mitsakakis K. (2018). Temperature change rate actuated bubble mixing for homogeneous rehydration of dry pre-stored reagents in centrifugal microfluidics. Lab Chip.

[37] Mark D., Weber P., Lutz S., Focke M., Zengerle R., von Stetten F. (2011). Aliquoting on the centrifugal microfluidic platform based on centrifugo-pneumatic valves. Microfluid. Nanofluid..

[38] Focke M., Stumpf F., Faltin B., Reith P., Bamarni D., Wadle S., Müller C., Reinecke H., Schrenzel J., Francois P. (2010). Microstructuring of polymer films for sensitive genotyping by real-time PCR on a centrifugal microfluidic platform. Lab Chip.

[39] Focke M., Kosse D., Al-Bamerni D., Lutz S., Müller C., Reinecke H., Zengerle R., von Stetten F. (2011). Microthermoforming of microfluidic substrates by soft lithography (µTSL): Optimization using design of experiments. J. Micromech. Microeng..

[40] Hahn-Schickard, Lab-on-a-Chip Foundry Service. https://www.hahn-schickard.de/en/production/lab-on-a-chip-foundry.

[41] Rombach M., Kosse D., Faltin B., Wadle S., Roth G., Zengerle R., von Stetten F. (2014). Real-time stability testing of air-dried primers and fluorogenic hydrolysis probes stabilized by trehalose and xanthan. BioTechniques.

[42] Hin S., Baumgartner D., Specht M., Lüddecke J., Arjmand E.M., Johannsen B., Schiedel L., Rombach M., Paust N., von Stetten F. (2020). VectorDisk: A Microfluidic Platform Integrating Mosquito Vector Markers for Evidence Based Control Applications. Processes.

[43] R Core Team (2015). R: A Language and Environment for Statistical Computing.

[44] Wickham H., Averick M., Bryan J., Chang W., D’Agostino McGowan L., François R., Grolemund G., Hayes A., Henry L., Hester J. (2019). Welcome to the tidyverse. J. Open Source Softw..

[45] Mitsakakis K., D’Acremont V., Hin S., von Stetten F., Zengerle R. (2018). Diagnostic tools for tackling febrile illness and enhancing patient management. Microelectron. Eng..

[46] Righolt A., Jevdejvic M., Marcenes W., Listl S. (2018). Global-, Regional-, and Country-Level Economic Impacts of Dental Diseases in 2015. J. Dent. Res..

[47] Cullinan M.P., Ford P.J., Seymour G.J. (2009). Periodontal disease and systemic health: Current status. Aust. Dent. J..

[48] Seymour G.J., Ford P.J., Cullinan M.P., Leishman S., Yamazaki K. (2007). Relationship between periodontal infections and systemic disease. Clin. Microbiol. Infect..

[49] Grigoriadis A., Sorsa T., Raisanen I., Parnanen P., Tervahartiala T., Sakellari D. (2019). Prediabetes/Diabetes Can Be Screened at the Dental Office by a Low-Cost and Fast Chair-Side/Point-of-Care aMMP-8 Immunotest. Diagnostics.

[50] Smits K.P.J., Listl S., Plachokova A.S., Van der Galien O., Kalmus O. (2020). Effect of periodontal treatment on diabetes-related healthcare costs: A retrospective study. BMJ Open Diab. Res. Care.

[51] Website of Dentognostics GmbH. https://www.dentognostics.de/en/.

[52] Leppilahti J.M., Ahonen M.M., Hernandez M., Munjal S., Netuschil L., Uitto V.J., Sorsa T., Mantyla P. (2011). Oral rinse MMP-8 point-of-care immuno test identifies patients with strong periodontal inflammatory burden. Oral Dis..

[53] Borujeni S.I., Mayer M., Eickholz P. (2015). Activated matrix metalloproteinase-8 in saliva as diagnostic test for periodontal disease? A case-control study. Med. Microbiol. Immunol..

[54] Al-Majid A., Alassiri S., Rathnayake N., Tervahartiala T., Gieselmann D.R., Sorsa T. (2018). Matrix Metalloproteinase-8 as an Inflammatory and Prevention Biomarker in Periodontal and Peri-Implant Diseases. Int. J. Dent..

[55] Website of OralDNA Labs, MyPerioPath®. https://www.oraldna.com/test/myperiopath/.

[56] Website of Direct Diagnostics. https://www.directdiagnostics.com/hr5.

[57] Website of PerioPOC®. https://en.periopoc.com/.

[58] Arweiler N.B., Marx V.K., Laugisch O., Sculean A., Auschill T.M. (2019). Clinical evaluation of a newly developed chairside test to determine periodontal pathogens. J. Periodontol..

[59] Bellagambi F.G., Lomonaco T., Salvo P., Vivaldi F., Hangouet M., Ghimenti S., Biagini D., Di Francesco F., Fuoco R., Errachid A. (2020). Saliva sampling: Methods and devices. An overview. TrAC Trends Anal. Chem..

[60] Khurshid Z., Zohaib S., Najeeb S., Zafar M.S., Slowey P.D., Almas K. (2016). Human Saliva Collection Devices for Proteomics: An Update. Int. J. Mol. Sci..

[61] Khanna P., Walt D.R. (2015). Salivary diagnostics using a portable point-of-service platform: A Review. Clin. Ther..

[62] Williams E., Bond K., Zhang B., Putland M., Williamson D.A. (2020). Saliva as a Noninvasive Specimen for Detection of SARS-CoV-2. J. Clin. Microbiol..

[63] To K.K.-W., Tsang O.T.-Y., Yip C.C.-Y., Chan K.-H., Wu T.-C., Chan J.M.-C., Leung W.-S., Chik T.S.-H., Choi C.Y.-C., Kandamby D.H. (2020). Consistent Detection of 2019 Novel Coronavirus in Saliva. Clin. Infect. Dis..

[64] Jacobs R., Maasdorp E., Malherbe S., Loxton A.G., Stanley K., van der Spuy G., Walzl G., Chegou N.N. (2016). Diagnostic Potential of Novel Salivary Host Biomarkers as Candidates for the Immunological Diagnosis of Tuberculosis Disease and Monitoring of Tuberculosis Treatment Response. PLoS ONE.

[65] Khan R.S., Khurshid Z., Asiri F.Y.I. (2017). Advancing Point-of-Care (PoC) Testing Using Human Saliva as Liquid Biopsy. Diagnostics.

[66] Rathnayake N., Gieselmann D.R., Heikkinen A.M., Tervahartiala T., Sorsa T. (2017). Salivary Diagnostics: Point-of-Care diagnostics of MMP-8 in dentistry and medicine. Diagnostics.

[67] Ji S., Choi Y. (2015). Point-of-care diagnosis of periodontitis using saliva: Technically feasible but still a challenge. Front. Cell. Infect. Microbiol..

[68] Johannsen B., Müller L., Baumgartner D., Karkossa L., Fruh S.M., Bostanci N., Karpisek M., Zengerle R., Paust N., Mitsakakis K. (2019). Automated Pre-Analytic Processing of Whole Saliva Using Magnet-Beating for Point-of-Care Protein Biomarker Analysis. Micromachines.

[69] De Boer R., Peters R., Gierveld S., Schuurman T., Kooistra-Smid M., Savelkoul P. (2010). Improved detection of microbial DNA after bead-beating before DNA isolation. J. Microbiol. Methods.

[70] Li X.L., Bosch-Tijhof C.J., Wei X., de Soet J.J., Crielaard W., van Loveren C., Deng D.M. (2020). Efficiency of chemical versus mechanical disruption methods of DNA extraction for the identification of oral Gram-positive and Gram-negative bacteria. J. Int. Med. Res..

[71] Larsen T., Fiehn N.E. (2017). Dental biofilm infections—An update. Apmis.

[72] Belibasakis G.N., Bostanci N., Marsh P.D., Zaura E. (2019). Applications of the oral microbiome in personalized dentistry. Arch. Oral Biol..

[73] Paqué P.N., Herz C., Wiedemeier D.B., Mitsakakis K., Attin T., Bao K., Belibasakis G.N., Hays J.P., Jenzer J.S., Kaman W.E. (2021). Salivary Biomarkers for Dental Caries Detection and Personalized Monitoring. J. Pers. Med..

[74] Zhao Y., Czilwik G., Klein V., Mitsakakis K., Zengerle R., Paust N. (2017). C-reactive protein and interleukin 6 microfluidic immunoassays with on-chip pre-stored reagents and centrifugo-pneumatic liquid control. Lab Chip.

[75] Johannsen J., Karpíšek M., Baumgartner D., Klein V., Bostanci N., Paust N., Früh S.M., Zengerle R., Mitsakakis K. (2021). One-step, wash-free, bead-based immunoassay employing bound-free phase detection. Anal. Chim. Acta.

[76] Mitsakakis K. (2021). Novel lab-on-a-disk platforms: A powerful tool for molecular fingerprinting of oral and respiratory tract infections. Expert Rev. Mol. Diagn..

[77] Seitz M.W., Haux C., Smits K.P.J., Kalmus O., Van Der Zande M.M., Lutyj J., Listl S. (2021). Development and evaluation of a mobile patient application to enhance medical-dental integration for the treatment of periodontitis and diabetes. Int. J. Med. Inform..

