# Supplementary material for: OralDisk: A Chair-Side Compatible Molecular Platform Using Whole Saliva for Monitoring Oral Health at the Dental Practice
Source: Biosensors (Basel). 2021 Oct 28;11(11):423. doi: 10.3390/bios11110423 (PMC8615610; doi:10.3390/bios11110423)
Supplement: Supplementary file 1 [file biosensors-11-00423-s001.zip › biosensors-1425783-supplementary.pdf]

biosensors

MDPI

Supplementary Materials

OralDisk: A Chair-Side Compatible Molecular Platform Using Whole Saliva for Monitoring Oral Health at the Dental Practice

Desirée Baumgartner [1,2,*], Benita Johannsen [1], Mara Specht [1], Jan Lüddecke [1], Markus Rombach [1], Sebastian Hin [1], Nils Paust [1,2], Felix von Stetten [1,2], Roland Zengerle [1,2], Christopher Herz [3], Johannes R. Peham [3], Pune N. Paqué [4], Thomas Attin [4], Joël S. Jenzer [4], Philipp Körner [4], Patrick R. Schmidlin [4], Thomas Thurnheer [4], Florian J. Wegehaupt [4], Wendy E. Kaman [5,6], Andrew Stubbs [7], John P. Hays [5], Viorel Rusu [8], Alex Michie [9], Thomas Binsl [9], David Stejskal [10,11], Michal Karpíšek [12,13], Kai Bao [14], Nagihan Bostanci [14], Georgios N. Belibasakis [14] and Konstantinos Mitsakakis [1,2,*]

[1] Hahn-Schickard, Georges-Koehler-Allee 103, 79110 Freiburg, Germany; Benita.Johannsen@Hahn-Schickard.de (B.J.); Mara.Specht@Hahn-Schickard.de (M.S.); Jan.Lueddecke@Hahn-Schickard.de (J.L.); Markus.Rombach@Hahn-Schickard.de (M.R.); sebastian.hin@iuvas.de (S.H.); Nils.Paust@Hahn-Schickard.de (N.P.); Felix.von.Stetten@Hahn-Schickard.de (F.v.S.); Roland.Zengerle@Hahn-Schickard.de (R.Z.)
[2] Laboratory for MEMS Applications, IMTEK–Department of Microsystems Engineering, University of Freiburg, Georges-Koehler-Allee 103, 79110 Freiburg, Germany
[3] AIT Austrian Institute of Technology, Molecular Diagnostics, Giefinggasse 4, 1210 Wien, Austria; christopher_herz@pall.com (C.H.); Johannes.Peham@ait.ac.at (J.R.P.)
[4] Clinic of Conservative and Preventive Dentistry, Center of Dental Medicine, University of Zurich, Plattenstrasse 11, 8032 Zurich, Switzerland; punenina.paque@zzm.uzh.ch (P.N.P.); thomas.attin@zzm.uzh.ch (T.A.); Joel.Jenzer@icloud.com (J.S.J.); philipp.koerner@zzm.uzh.ch (P.K.); patrick.schmidlin@zzm.uzh.ch (P.R.S.); Thomas.Thurnheer@zzm.uzh.ch (T.T.); florian.wegehaupt@zzm.uzh.ch (F.J.W.)
[5] Department of Medical Microbiology and Infectious Diseases, Erasmus University Medical Centre Rotterdam (Erasmus MC), 3015 CN Rotterdam, The Netherlands; w.e.kaman@acta.nl (W.E.K.); j.hays@erasmusmc.nl (J.P.H.)
[6] Department of Oral Biochemistry, Academic Centre for Dentistry Amsterdam (ACTA), Free University of Amsterdam and University of Amsterdam, 1081 LA Amsterdam, The Netherlands
[7] Department of Pathology and Clinical Bioinformatics, Erasmus University Medical Centre Rotterdam (Erasmus MC), 3015 CN Rotterdam, The Netherlands; a.stubbs@erasmusmc.nl
[8] magtivio B.V., Daelderweg 9, 6361 HK Nuth, The Netherlands; vru@magtivio.com
[9] ClinicaGeno Ltd., 11 Station Approach, Coulsdon, CR5 2NR, UK; alex@clinicageno.com (A.M.); thomas@clinicageno.com (T.B.)
[10] Department of Biomedical Sciences, Faculty of Medicine, University of Ostrava, Syllabova 19, 70300 Ostrava, Czech Republic; david.stejskal@fno.cz
[11] Institute of Laboratory Diagnostics, University Hospital Ostrava, 17. listopadu 1790/5, 70800 Ostrava, Czech Republic
[12] BioVendor-Laboratorní medicína a.s., Research & Diagnostic Products Division, Karasek 1767/1, Reckovice, 62100 Brno, Czech Republic; karpisek@biovendor.com
[13] Faculty of Pharmacy, Masaryk University, Palackeho trida 1946/1, 61242 Brno, Czech Republic
[14] Section of Oral Health and Periodontology, Division of Oral Diseases, Department of Dental Medicine, Karolinska Institutet, 14104 Huddinge, Sweden; kai.bao@ki.se (K.B.); nagihan.bostanci@ki.se (N.B.); george.belibasakis@ki.se (G.N.B.)
* Correspondence: Konstantinos.Mitsakakis@Hahn-Schickard.de (K.M.); Desiree.Baumgartner@Hahn-Schickard.de (D.B.); Tel.: +49-761-203-73252 (K.M.); +49-761-203-98724 (D.B.)

Citation: Baumgartner, D.; Johannsen, B.; Specht, M.; Lüddecke, J.; Rombach, M.; Hin, S.; Paust, N.; von Stetten, F.; Zengerle, R.; Herz, C.; et al. OralDisk: A Chair-Side Compatible Molecular Platform Using Whole Saliva for Monitoring Oral Health at the Dental Practice. *Biosensors* **2021**, *11*, x. https://doi.org/10.3390/xxxxx

Received: 1 October 2021
Accepted: 24 October 2021
Published: 28 October 2021

Publisher's Note: MDPI stays neutral with regard to jurisdictional claims in published maps and institutional affiliations.

Copyright: © 2021 by the authors. Submitted for possible open access publication under the terms and conditions of the Creative Commons Attribution (CC BY) license (https://creativecommons.org/licenses/by/4.0/).

Table S1. Raw experimental data (Cq values) from the OralDisk and the lab-based reference method, as well as qualitative data from the iai PadoTest. The latter includes only five bacteria common with the OralDisk panel, that is why the others are denoted with grey shading in the iai PadoTest section of the table. 'Ch.1-Ch10' refers to the numbering of OralDisk chambers, where each assay target was detected. The samples are coded with a long and short name. The short names starting with 'H', 'C' and 'P' represent the diagnosis group names 'healthy', 'caries' and 'periodontitis', respectively. 'ND' stands for 'not detected'. 'NT' stands for 'not tested'. 'D' stands for 'detected' (in the iai PadoTest section). The bold fonts in the lab-based reference and OralDisk sections of the table denote the deviation of the corresponding assay between the two methods.

		**Ch.1**	**Ch.2**	**Ch.3**	**Ch.4**	**Ch.5**	**Ch.6**	**Ch.7**	**Ch.8**	**Ch.9**	**Ch.10**
		A. actinomycetemcomitans	*C. rectus*	*F. nucleatum*	*P. intermedia*	*P. gingivalis*	*T. forsythia*	*T. denticola*	*Lactobacillus* **spp.**	*S. mutans*	*S. sobrinus*
Abbrev. sample code	Full sample code	**Cq values**									
**Lab-based reference method**											
H1	GTT33	ND	25.8	23.2	21.5	23.2	25.9	ND	22.1	29.2	ND
H2	GDB75	ND	23.0	**20.5**	26.6	23.0	25.6	**ND**	19.5	27.0	ND
H3	GPD78	ND	25.7	**21.8**	24.1	25.3	24.7	ND	ND	23.9	ND
H4	GRR52	ND	24.8	**21.6**	25.1	23.8	24.8	ND	**20.0**	24.3	ND
H5	GRA31	**28.0**	26.1	22.7	**24.5**	23.6	25.9	24.3	ND	22.7	ND
H6	GRW58	ND	22.9	24.0	25.6	26.9	25.6	ND	**ND**	28.6	ND
H7	GJT79	ND	26.3	23.9	26.5	24.1	26.1	ND	**ND**	30.9	ND
C1	KxTC22	ND	**25.2**	**20.4**	24.2	23.1	24.0	22.7	17.9	21.0	26.3
C2	KHS14	ND	24.1	**23.1**	26.2	24.0	26.4	ND	18.0	22.8	ND
C3	KMDS17	ND	25.6	23.6	24.7	28.9	26.4	24.7	ND	25.8	27.0
C4	KLB33	**29.2**	26.3	23.9	24.2	27.5	27.2	26.1	ND	25.7	27.2
C5	KHD34	ND	25.1	19.4	27.4	20.0	23.0	25.6	20.6	21.8	ND
C6	KxDT13	ND	26.6	21.4	23.3	25.3	24.2	24.0	ND	28.3	28.1
C7	KNE35	26.5	24.6	20.1	21.1	22.4	23.4	22.3	24.7	24.7	ND
C8	KxOD23	**26.9**	23.3	21.5	26.9	21.6	24.6	23.2	**ND**	26.5	ND
C9	KxPW03	ND	24.4	22.5	25.1	21.7	23.8	23.2	**ND**	18.5	**24.6**
P1	PJK22	ND	23.9	**21.2**	23.5	20.1	24.4	21.3	**20.9**	**28.0**	ND
P2	PBG38	ND	24.7	23.3	**27.4**	25.0	25.5	**ND**	ND	**31.3**	ND
P3	PMN25	ND	23.0	21.4	23.6	19.6	22.9	22.7	ND	23.8	ND
P4	PDE05	ND	24.4	23.0	26.5	26.2	27.4	22.5	**17.4**	24.8	ND
P5	PAM65	ND	23.1	21.5	20.3	20.9	24.0	20.8	**18.0**	24.5	24.7
P6	PMDK19	ND	**24.8**	**21.8**	22.8	22.7	24.3	23.6	ND	29.2	ND
P7	KxHS17	ND	27.8	23.0	23.7	24.8	27.5	ND	22.6	23.0	24.7
P8	KxFK05	ND	25.2	22.1	25.8	25.3	25.5	26.3	**ND**	26.5	ND
**OralDisk**											
H1	GTT33	ND	23.5	22.4	20.5	22.3	27.3	ND	25.0	24.4	ND
H2	GDB75	ND	23.8	**ND**	33.3	NT	NT	**25.4**	21.6	28.6	ND
H3	GPD78	ND	25.4	**ND**	30.0	NT	NT	ND	ND	27.1	ND
H4	GRR52	ND	20.2	**ND**	32.8	23.8	26.5	ND	**ND**	26.4	ND
H5	GRA31	**ND**	27.3	26.0	**ND**	22.5	23.9	28.1	ND	26.5	ND
H6	GRW58	ND	NT	NT	26.6	25.5	26.9	ND	**32.8**	31.7	ND
H7	GJT79	ND	26.2	27.0	28.4	23.3	28.1	ND	**35.3**	33.9	ND
C1	KxTC22	ND	**ND**	**ND**	18.5	19.5	20.5	20.1	20.7	24.4	24.6
C2	KHS14	ND	22.0	**ND**	25.2	21.8	25.5	ND	19.4	24.5	ND
C3	KMDS17	ND	23.0	23.2	21.5	28.5	25.2	25.0	ND	26.7	29.3
C4	KLB33	**ND**	25.7	24.0	23.3	26.3	27.2	24.5	ND	26.7	29.4
C5	KHD34	ND	26.7	20.4	35.7	21.2	25.3	27.0	30.7	29.6	ND
C6	KxDT13	ND	21.8	19.7	18.5	22.7	20.5	21.8	ND	31.5	30.0
C7	KNE35	27.7	23.6	20.2	21.4	21.2	23.3	23.3	21.9	28.4	ND
C8	KxOD23	**ND**	20.4	20.1	30.0	19.8	22.0	22.2	**27.0**	29.4	ND
C9	KxPW03	ND	25.7	20.4	24.2	18.9	23.1	21.7	**26.8**	33.4	**ND**
P1	PJK22	ND	20.3	**ND**	20.6	18.1	20.1	20.0	**ND**	**ND**	ND

P2	PBG38	ND	19.8	17.8	**ND**	19.0	20.0	**20.6**	ND	**ND**	ND
P3	PMN25	ND	17.9	18.6	22.4	19.3	20.6	20.3	ND	23.6	ND
P4	PDE05	ND	21.8	19.7	27.8	26.4	26.8	22.7	**ND**	24.9	ND
P5	PAM65	ND	22.0	21.7	23.4	21.9	21.5	24.5	**ND**	21.6	26.9
P6	PMDK19	ND	**ND**	**ND**	19.2	19.3	21.1	21.0	ND	27.2	ND
P7	KxHS17	ND	29.7	26.1	24.2	24.0	27.3	ND	25.1	25.7	30.8
P8	KxFK05	ND	25.9	24.0	28.2	25.9	24.9	28.5	**27.1**	29.7	ND
**iai PadoTest**											
H1	GTT33	ND			D	ND	ND	ND			
H2	GDB75	ND			ND	ND	ND	D			
H3	GPD78	ND			ND	ND	ND	ND			
H4	GRR52	ND			ND	ND	ND	ND			
H5	GRA31	ND			ND	ND	ND	D			
H6	GRW58	ND			ND	ND	ND	ND			
H7	GJT79	ND			ND	D	ND	ND			
C1	KxTC22	Sample not tested									
C2	KHS14	ND			D	ND	ND	ND			
C3	KMDS17	ND			ND	ND	ND	ND			
C4	KLB33	ND			D	ND	ND	D			
C5	KHD34	ND			ND	D	ND	D			
C6	KxDT13	Sample not tested									
C7	KNE35	ND			D	D	D	D			
C8	KxOD23	Sample not tested									
C9	KxPW03	Sample not tested									
P1	PJK22	ND			D	D	D	D			
P2	PBG38	ND			ND	ND	ND	D			
P3	PMN25	ND			D	D	D	D			
P4	PDE05	ND			ND	D	ND	D			
P5	PAM65	ND			D	D	ND	D			
P6	PMDK19	Sample not tested									
P7	KxHS17	Sample not tested									
P8	KxFK05	ND			ND	D	ND	ND			

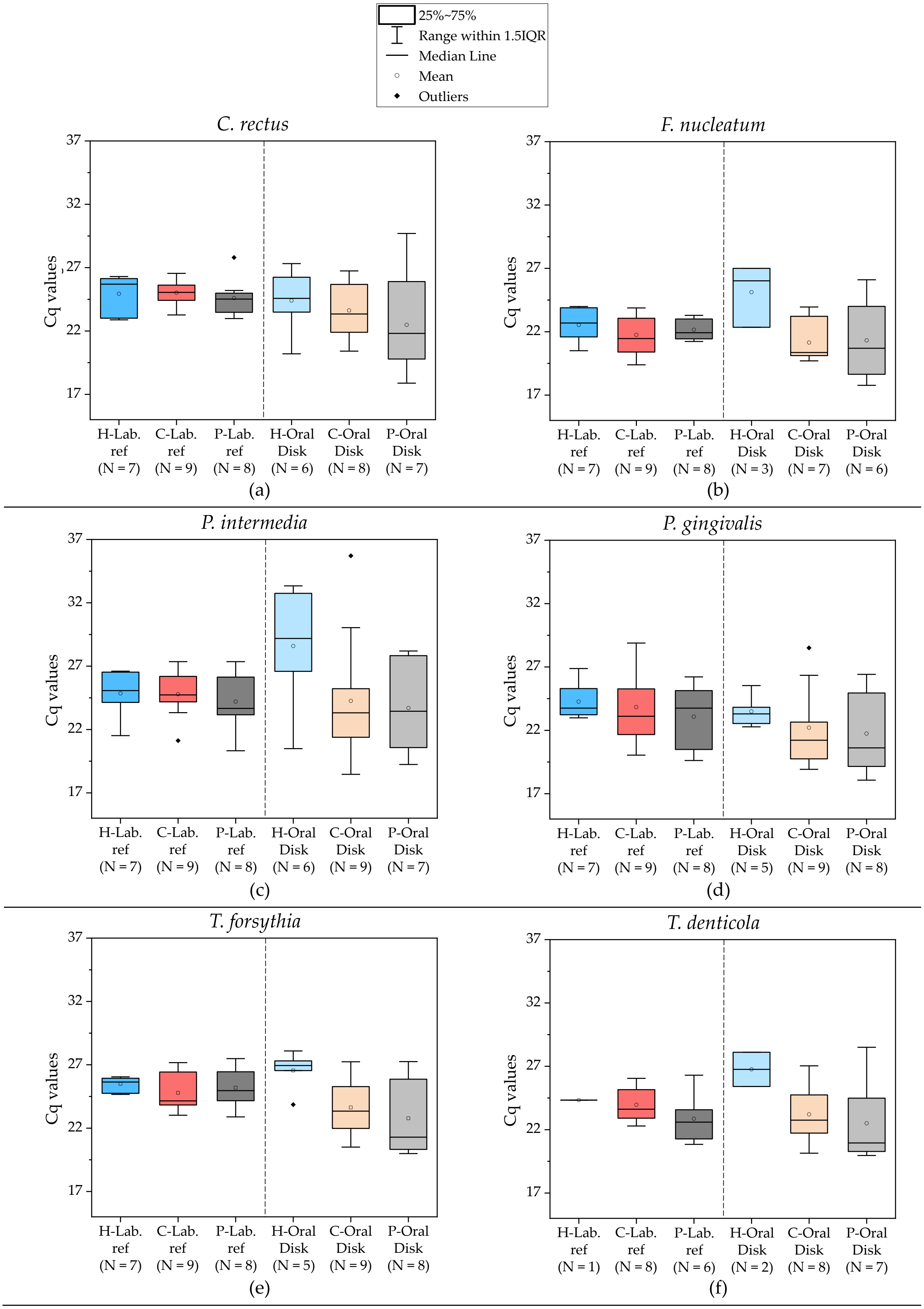
25%~75%
Range within 1.5IQR
Median Line
Mean
Outliers
C. rectus
F. nucleatum
P. intermedia
P. gingivalis
T. forsythia
T. denticola
Cq values
37
32
27
22
17
H-Lab. ref (N = 7)
C-Lab. ref (N = 9)
P-Lab. ref (N = 8)
H-Oral Disk (N = 6)
C-Oral Disk (N = 8)
P-Oral Disk (N = 7)
(a)
H-Lab. ref (N = 7)
C-Lab. ref (N = 9)
P-Lab. ref (N = 8)
H-Oral Disk (N = 3)
C-Oral Disk (N = 7)
P-Oral Disk (N = 6)
(b)
H-Lab. ref (N = 7)
C-Lab. ref (N = 9)
P-Lab. ref (N = 8)
H-Oral Disk (N = 6)
C-Oral Disk (N = 9)
P-Oral Disk (N = 7)
(c)
H-Lab. ref (N = 7)
C-Lab. ref (N = 9)
P-Lab. ref (N = 8)
H-Oral Disk (N = 5)
C-Oral Disk (N = 9)
P-Oral Disk (N = 8)
(d)
H-Lab. ref (N = 7)
C-Lab. ref (N = 9)
P-Lab. ref (N = 8)
H-Oral Disk (N = 5)
C-Oral Disk (N = 9)
P-Oral Disk (N = 8)
(e)
H-Lab. ref (N = 1)
C-Lab. ref (N = 8)
P-Lab. ref (N = 6)
H-Oral Disk (N = 2)
C-Oral Disk (N = 8)
P-Oral Disk (N = 7)
(f)

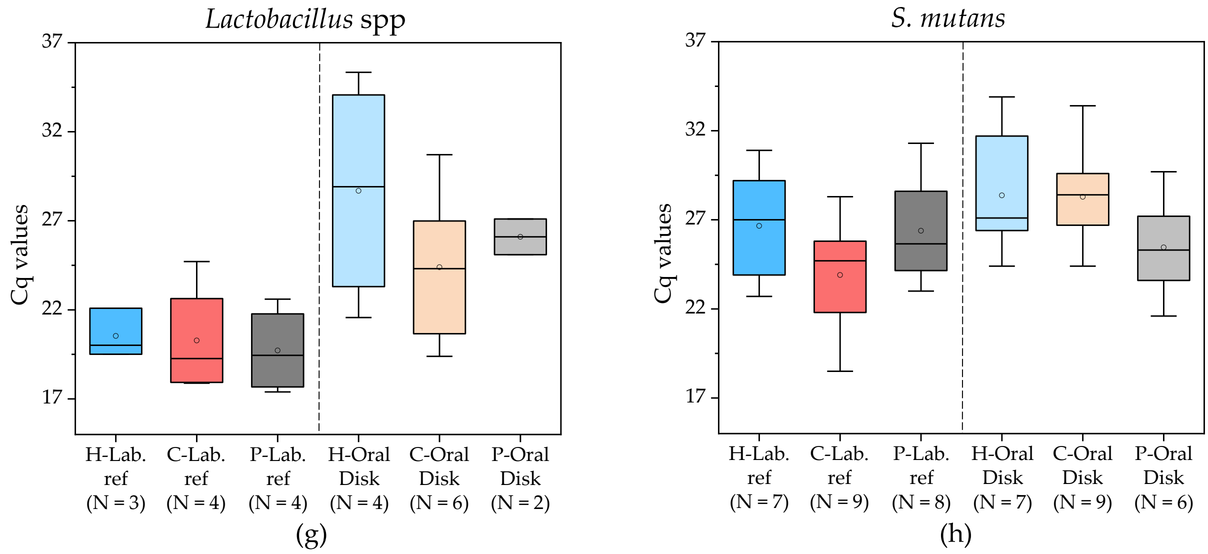

Figure S1. Boxplots of the Cq values for each bacterium detected by the OralDisk and the lab-based reference: (**a**) *C. rectus*; (**b**) *F. nucleatum*; (**c**) *P. intermedia*; (**d**) *P. gingivalis*; (**e**) *T. forsythia*; (**f**) *T. denticola*; (**g**) *Lactobacillus* spp.; (**h**) *S. mutans*. The bacteria *A. actinomycetemcomitans* and *S. sobrinus* are not included because of insufficient measurement points to derive boxplots (Supplementary Table S2). The number (N) under each boxplot denotes the number of positive assay targets detected by the specific test method for the specific group and bacterium. The dashed line in each set separates the boxplots of the three diagnosis groups tested with the lab-based reference versus the OralDisk.

Table S2. Median and interquartile range (IQR, describing the middle 50% of values when ordered from lowest to highest) of the Cq values from the OralDisk and lab-based reference measurements. 'Ch.1-Ch10' refers to the numbering of the OralDisk chambers, where each assay target was detected. The number (N_{pos}/N_{test}) denotes the number of positive assay targets over the total tested by the specific method for the specific group and bacterium. 'N/A' means that statistical analysis was not possible due to less than three measurements. The abbreviations 'H', 'C' and 'P' represent the diagnosis group names 'healthy', 'caries' and 'periodontitis', respectively.

			**Ch.1**	**Ch.2**	**Ch.3**	**Ch.4**	**Ch.5**	**Ch.6**	**Ch.7**	**Ch.8**	**Ch.9**	**Ch.10**
			A. actinomycetemcomitans	***C. rectus***	***F. nucleatum***	***P. intermedia***	***P. gingivalis***	***T. forsythia***	***T. denticola***	***Lactobacillus* spp.**	***S. mutans***	***S. sobrinus***
H	OralDisk	Median	N/A	24.6	26.0	29.2	23.3	26.9	26.8	28.9	27.1	N/A
		IQR	N/A	2.5	2.3	5.0	1.3	0.8	1.4	9.3	3.7	N/A
		(N_{pos}/N_{test})	(0/7)	(6/6) [a]	(3/6) [a]	(6/7)	(5/5) [a]	(5/5) [a]	(2/7)	(4/7)	(7/7)	(0/7)
H	Lab-reference	Median	28.0	25.7	22.7	25.1	23.8	25.6	24.3	20.0	27.0	N/A
		IQR	N/A	2.1	1.8	1.7	1.3	0.7	N/A	1.3	4.8	N/A
		(N_{pos}/N_{test})	(1/7)	(7/7)	(7/7)	(7/7)	(7/7)	(7/7)	(1/7)	(3/7)	(7/7)	(0/7)
C	OralDisk	Median	N/A	23.3	20.4	23.3	21.2	23.3	22.8	24.3	28.4	29.4
		IQR	N/A	3.7	1.6	3.8	2.9	3.3	2.9	6.0	2.9	1.4
		(N_{pos}/N_{test})	(1/9)	(8/9)	(7/9)	(9/9)	(9/9)	(9/9)	(8/9)	(6/9)	(9/9)	(4/9)
C	Lab-reference	Median	26.9	25.1	21.5	24.7	23.1	24.2	23.6	19.3	24.7	27.0
		IQR	1.3	1.2	2.7	2.0	3.6	2.6	1.9	3.7	4.0	0.9
		(N_{pos}/N_{test})	(3/9)	(9/9)	(9/9)	(9/9)	(9/9)	(9/9)	(8/9)	(4/9)	(9/9)	(5/9)
P	OralDisk	Median	N/A	21.8	20.7	23.4	20.6	21.3	21.0	26.1	25.3	28.9
		IQR	N/A	3.9	4.5	4.6	5.3	5.0	3.2	1.0	2.9	2.0
		(N_{pos}/N_{test})	(0/8)	(7/8)	(6/8)	(7/8)	(8/8)	(8/8)	(7/8)	(2/8)	(6/8)	(2/8)
P	Lab-reference	Median	N/A	24.5	21.9	23.7	23.8	24.9	22.6	19.4	25.6	24.7
		IQR	N/A	1.2	1.6	2.6	4.4	1.7	1.8	3.5	4.0	0.0
		(N_{pos}/N_{test})	(0/8)	(8/8)	(8/8)	(8/8)	(8/8)	(8/8)	(6/8)	(4/8)	(8/8)	(2/8)

[a] In one disk, signals of *C. rectus* and *F. nucleatum* where not measured, while in two (other) disks, signals of *P. gingivalis* and *T. forsythia* were not measured.

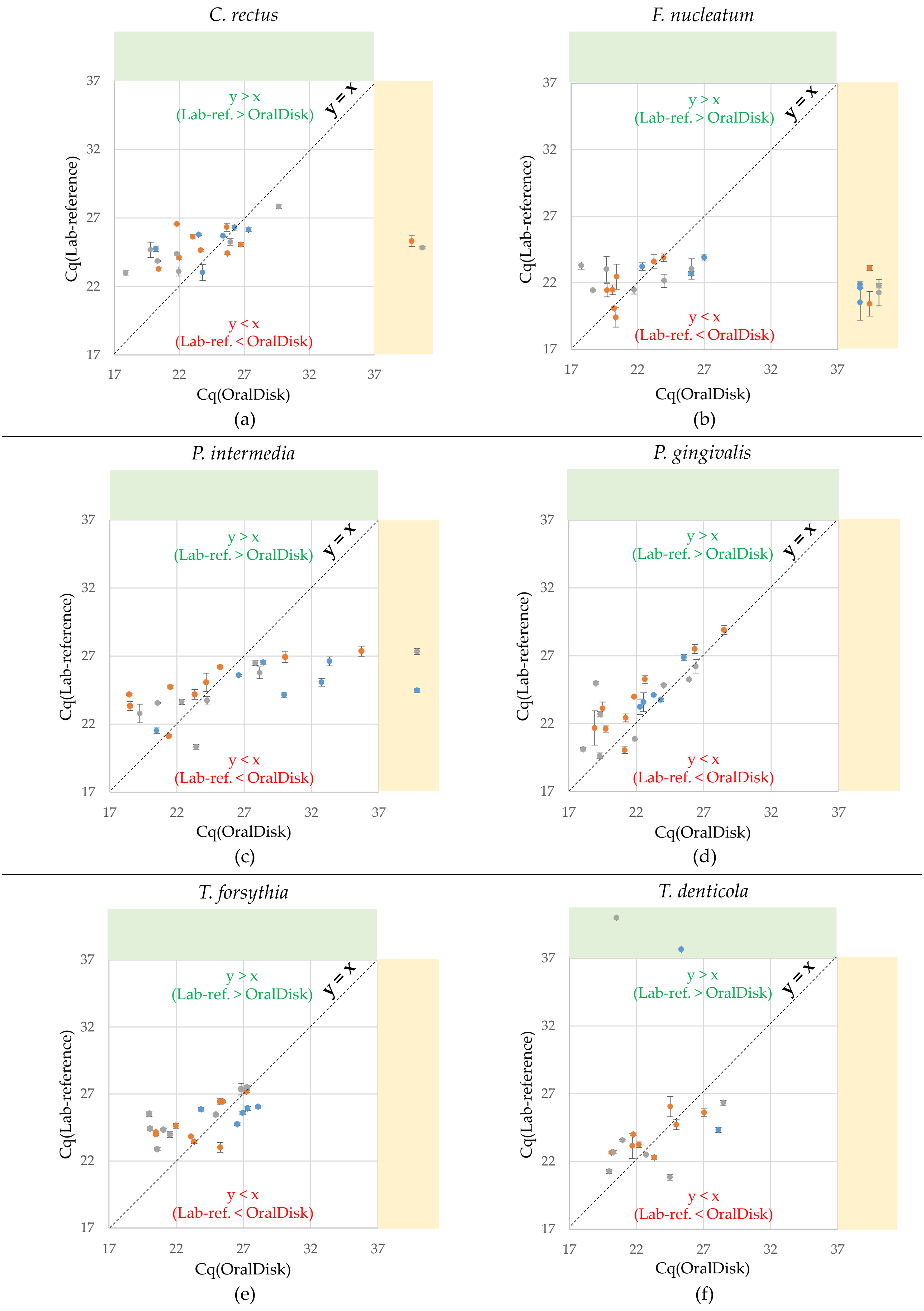
C. rectus
F. nucleatum
P. intermedia
P. gingivalis
T. forsythia
T. denticola
y > x
(Lab-ref. > OralDisk)
y < x
(Lab-ref. < OralDisk)
y = x
Cq(Lab-reference)
Cq(OralDisk)
17
22
27
32
37
(a)
(b)
(c)
(d)
(e)
(f)

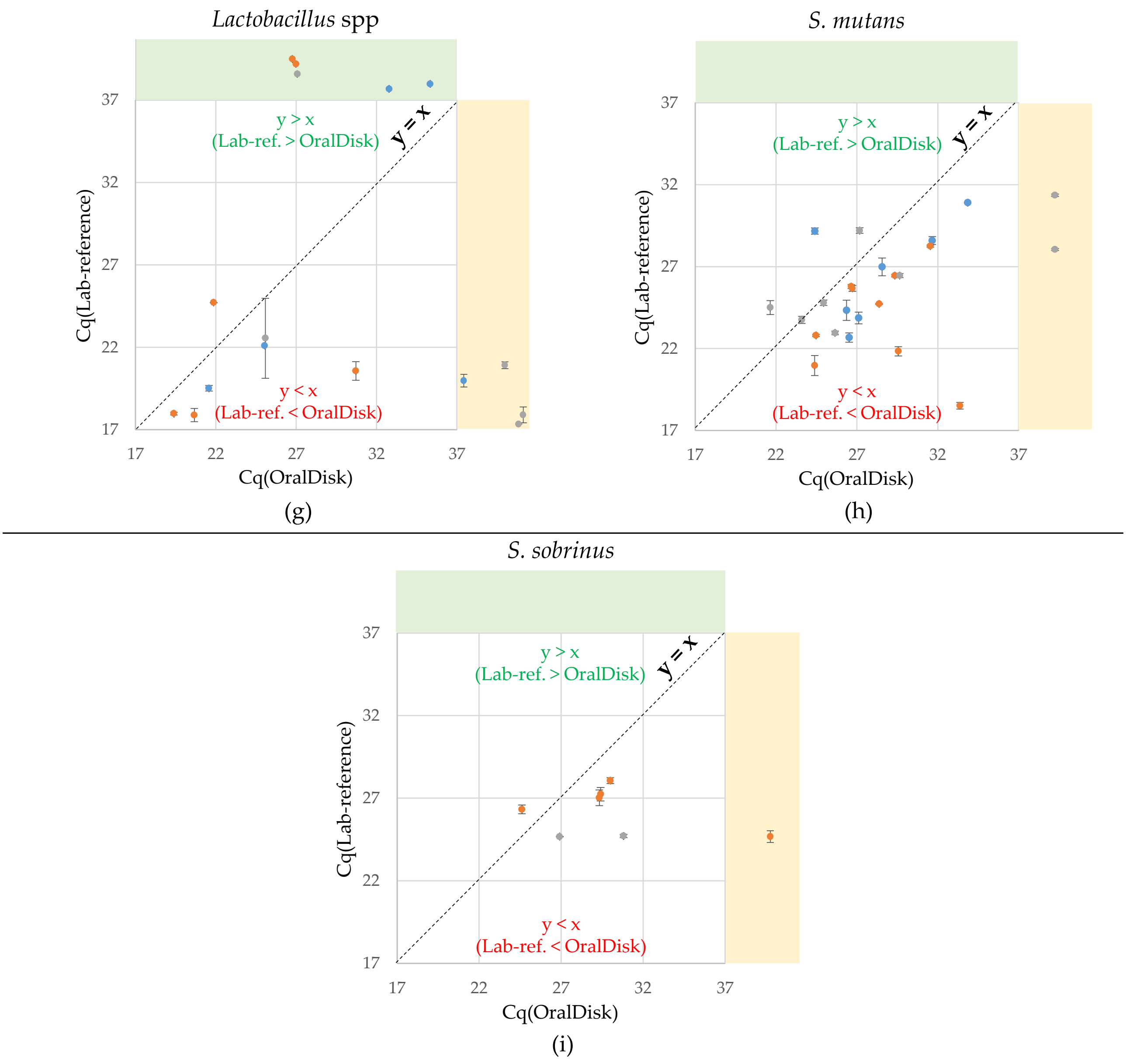

Figure S2. Scatter plots correlating the Cq values from the lab-based reference and the OralDisk for all bacteria in the healthy (●), caries (●) and periodontitis (●) groups: (**a**) *C. rectus*; (**b**) *F. nucleatum*; (**c**) *P. intermedia*; (**d**) *P. gingivalis*; (**e**) *T. forsythia*; (**f**) *T. denticola*; (**g**) *Lactobacillus* spp.; (**h**) *S. mutans*; (**i**) *S. sobrinus*. The *A. actinomycetemcomitans* bacterium is not shown because there was only one data point common between the OralDisk and the lab-based reference. For data points above and below the y = x (dashed) line it means that Cq(Lab-reference) > Cq(OralDisk) and Cq(OralDisk) > Cq(Lab-reference), respectively. Assays that were detected only by one of the two methods are represented in the designated green (OralDisk) and orange (lab-based reference) zones, respectively, outside the scatter plots. Standard deviations were not possible to be included for the x-axis measurements (OralDisk PCR) as each sample was tested with only one OralDisk cartridge.
